# Serum and tissue markers in hepatocellular carcinoma and cholangiocarcinoma: clinical and prognostic implications

**DOI:** 10.18632/oncotarget.13929

**Published:** 2016-12-14

**Authors:** Massimiliano Berretta, Carla Cavaliere, Lara Alessandrini, Brigida Stanzione, Gaetano Facchini, Luca Balestreri, Tiziana Perin, Vincenzo Canzonieri

**Affiliations:** ^1^ Department of Medical Oncology, National Cancer Institute, Aviano (PN), Italy; ^2^ Department of Onco-Ematology Medical Oncology, S.G. Moscati Hospital of Taranto Taranto, Italy; ^3^ Division of Pathology, National Cancer Institute, Aviano (PN), Italy; ^4^ Department of Medical Oncology, National Cancer Institute, “G. Pascale” Foundation, Naples, Italy; ^5^ Department of Radiology, National Cancer Institute, Aviano (PN), Italy

**Keywords:** hepatocellular carcinoma, cholangiocarcinoma, serum markers, tissue markers, prognosis, metastasis

## Abstract

HCC represents the sixth most common cancer worldwide and the second leading cause of cancer-related death. Despite the high incidence, treatment options for advanced HCC remain limited and unsuccessful, resulting in a poor prognosis. Despite the major advances achieved in the diagnostic management of HCC, only one third of the newly diagnosed patients are presently eligible for curative treatments. Advances in technology and an increased understanding of HCC biology have led to the discovery of novel biomarkers. Improving our knowledge about serum and tissutal markers could ultimately lead to an early diagnosis and better and early treatment strategies for this deadly disease. Serum biomarkers are striking potential tools for surveillance and early diagnosis of HCC thanks to the non-invasive, objective, and reproducible assessments they potentially enable. To date, many biomarkers have been proposed in the diagnosis of HCC. Cholangiocarcinoma (CCA) is an aggressive malignancy, characterized by early lymph node involvement and distant metastasis, with 5-year survival rates of 5%-10%. The identification of new biomarkers with diagnostic, prognostic or predictive value is especially important as resection (by surgery or combined with a liver transplant) has shown promising results and novel therapies are emerging. However, the relatively low incidence of CCA, high frequency of co-existing cholestasis or cholangitis (primary sclerosing cholangitis –PSC- above all), and difficulties with obtaining adequate samples, despite advances in sampling techniques and in endoscopic visualization of the bile ducts, have complicated the search for accurate biomarkers. In this review, we attempt to analyze the existing literature on this argument.

## HEPATOCELLULAR CARCINOMA

## INTRODUCTION

Hepatocellular carcinoma (HCC) accounts as the sixth most common neoplasm on a global scale, and the third most lethal with >600,000 deaths per year worldwide [1, 2]. Despite the major advances achieved in the diagnostic workup of HCC, only one third of the newly diagnosed patients are presently eligible for curative treatments [3]. Even in the curative setting, 5-year survival rates after resection for early-stage HCC ranges between 17% and 53%, and recurrence rates can be as high as 70% [4, 5]. In patients treated with liver transplantation, overall survival (OS) rates approach 75% at 4 years and recurrence occurs in 8% to 15% of all graft recipients fulfilling the Milan criteria [6, 7]. In patients with unresectable disease, overall life expectancy is not homogeneously distributed and as this patient group is the current focus for clinical studies involving molecular targeted therapies, there is a need to predict prognosis before treatment [8, 9].

Major risk factors for HCC include HBV, HCV, diabetes, obesity, excess alcohol consumption and metabolic diseases. All these factors contribute to a perpetual state of inflammation and fibrogenesis, leading to fibrosis and cirrhosis, preneoplastic conditions promoting the development of HCC. In particular, most patients with chronic hepatitis will develop liver cirrhosis and eventually HCC in a progressive and dynamic process, thanks to an altered liver microenvironment characterized by the generation of highly reactive oxygen species and a constitutively active inflammatory milieu. Improving our knowledge about serum and tissue markers could ultimately lead to an early diagnosis and better and early treatment strategies for this deadly disease [10–12]. In this review, we attempt to analyze the existing literature on this argument.

## TISSUE MARKERS

As classical pathological and phenotypical parameters are only partly able to predict clinical behavior of individual tumors, new molecular tests and methods will have to be added into the morphology-based diagnostic procedure. Formalin-fixed paraffin-embedded (FFPE) tissue is still the most widely used method for tissue preparation in the routine diagnostic setting. In the past, extracting nucleic acids or proteins for molecular analysis from FFPE tissue has been difficult to perform. Nowadays, if the tissue is timely preserved in 10% neutral buffered formalin (final concentration: 4%) and if standard processing procedures are met, DNA and RNA of sufficient good quality for molecular analyses can easily be acquired [13].

### Diagnostic tissue markers

An International Consensus has recently been obtained on the classification of small (≤ 2 cm) hepatocellular nodules [14]. Nodular lesions found in chronic liver disease are classified into large regenerative nodule (LRN), low-grade dysplastic nodule (LGDN), high-grade dysplastic nodule (HGDN), and HCC. The most difficult differential diagnosis is with HGDN. Morphology alone is often not sufficient and requires additional techniques (Table 1).

**Figure 1:**
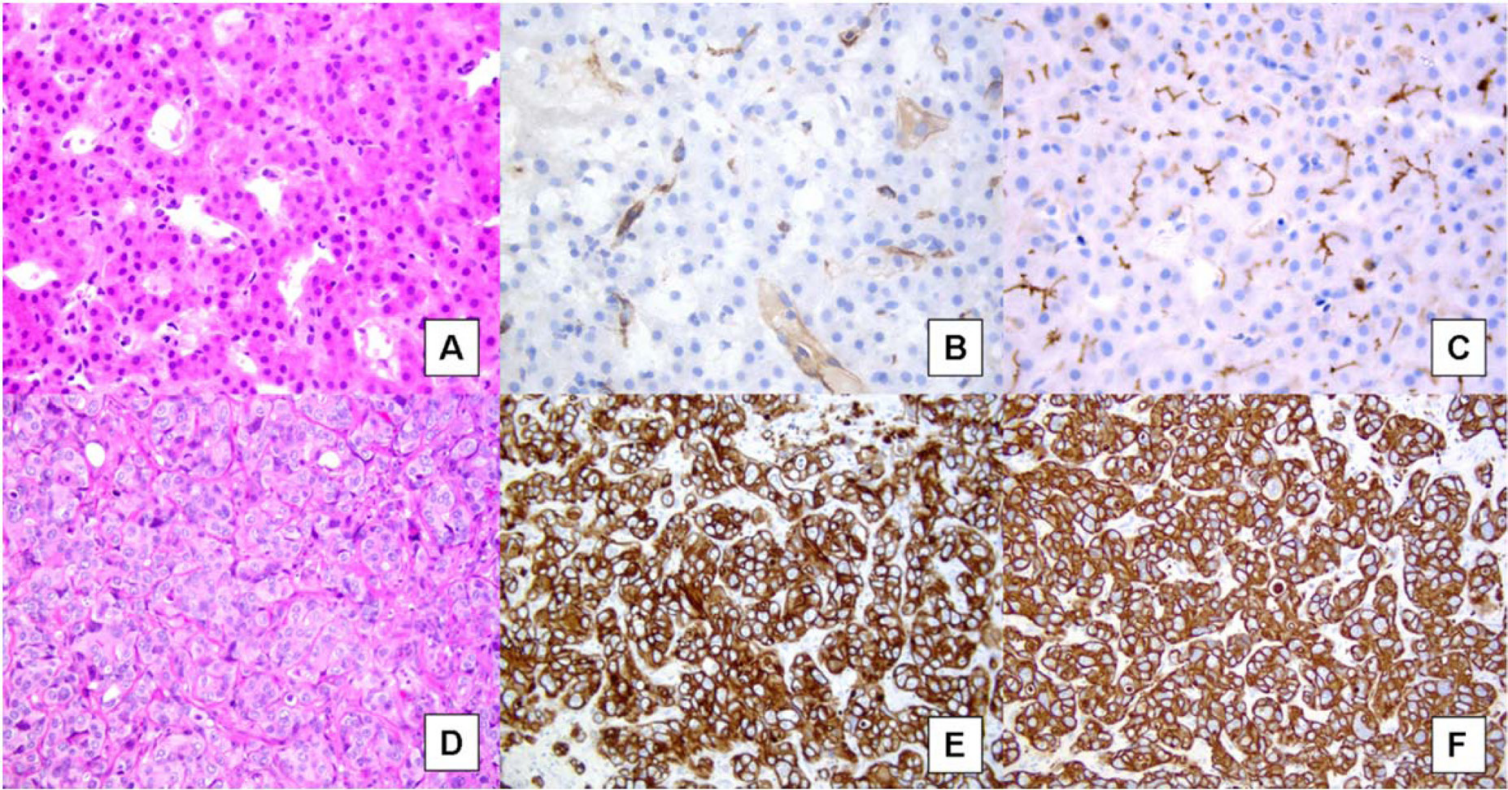
**A.** Well-differentiated hepatocellular carcinoma, showing a trabecular pattern (Hematoxylin & eosin). **B**. CD34 immunostaining highlights capillarization of sinusoids, with complete staining around vessel wall. **C**. CD10 immunostaining shows a peculiar canalicular pattern in HCC. **D**. Well-moderately differentiated cholangiocarcinoma growing in a trabecular pattern, similar to HCC (Hematoxylin & eosin). **E**. Strong and diffuse positivity for CK7. **F**. Strong and diffuse positivity for CK19.

**Figure 2:**
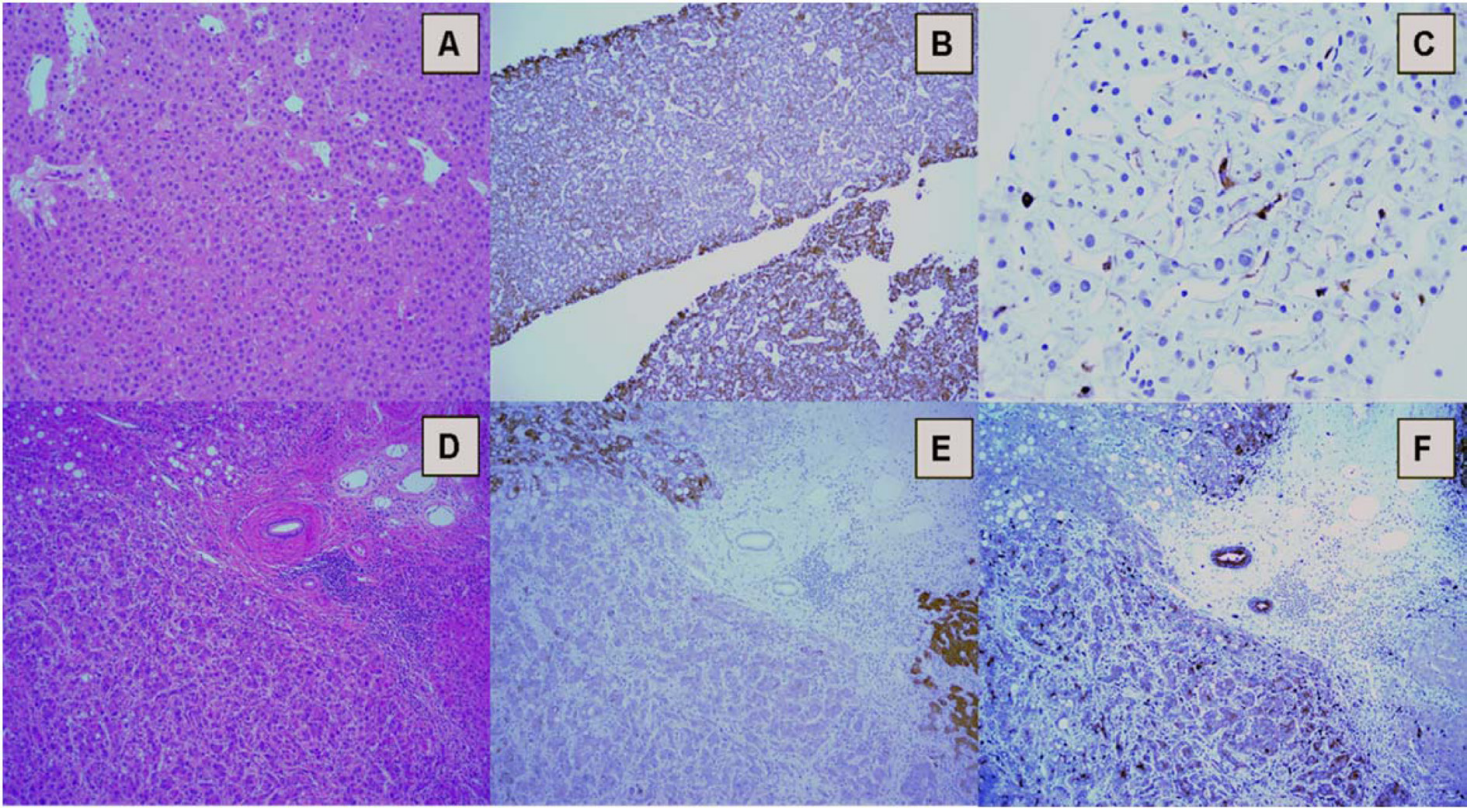
**A.** Biopsy of a well-differentiated hepatocellular carcinoma, showing a trabecular pattern (Hematoxylin & eosin). **B**. Hep Par 1 diffuse cytoplasmic granular staining. **C**. “Canalicular”pattern of pCEA immunostaining.**D**. Poorly differentiated HCC (surgical sample) with lipogenic differentiation. A portal tract is evident in the center. **E**. Hep Par 1 immunostaining is patchy, with areas showing strong and diffuse cytoplasmic reaction (top right) and areas with loss of staining (bottom). Normal hepatocytes (bottom right) are evident. Biliary epithelium and inflammatory cell in the portal tract are negative. **F**. pCEA immunostaining is cytoplasmic, uneven, and has lost the peculial canalicular pattern of C.

**Figure 3:**
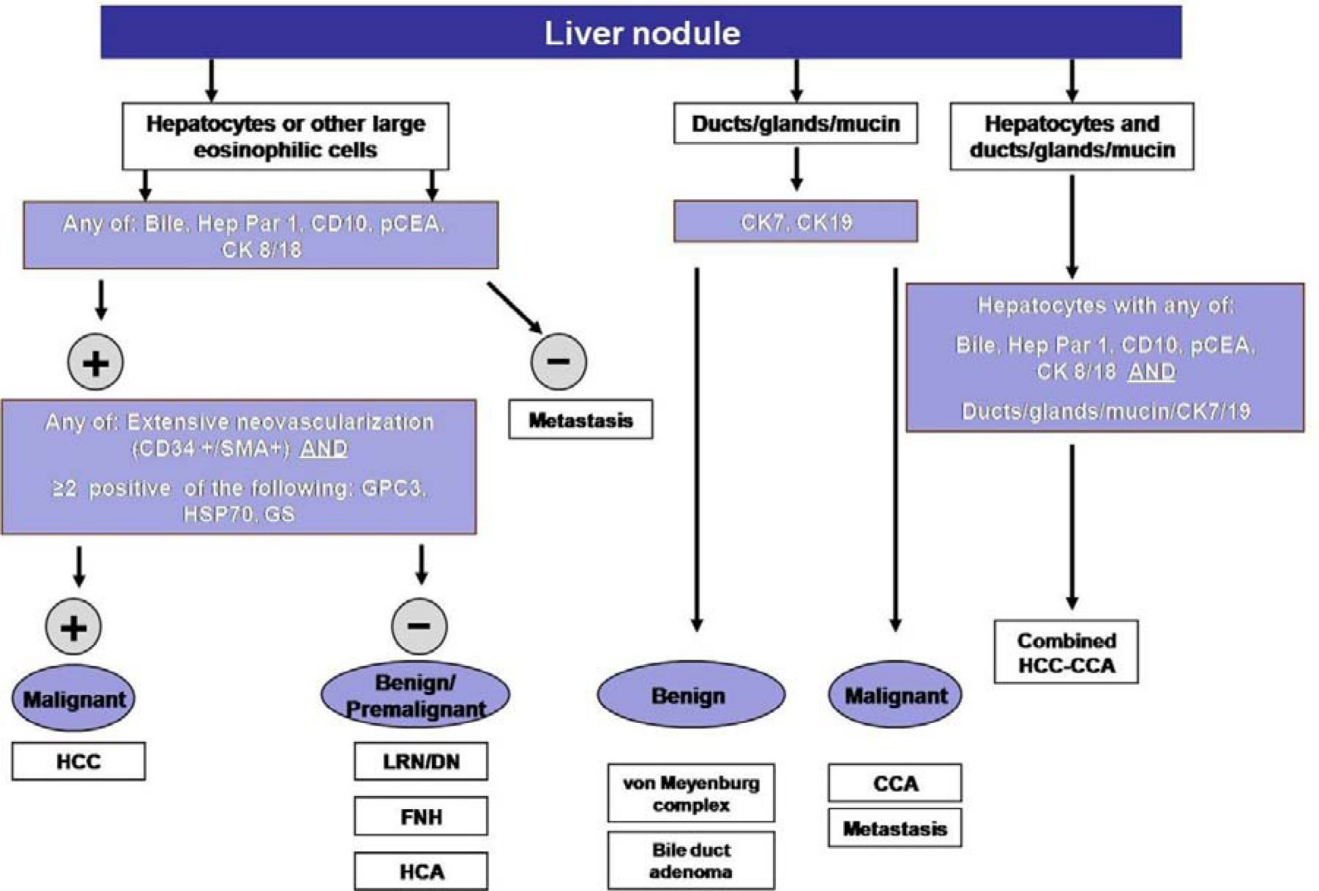
Simplified diagnostic algorithm in the pathologic evaluation of liver nodules

Extensive neovascularization process and sinusoids capillarization could be detected by immunohistochemical staining with endothelial marker CD34 (Figure 1A-B) and the Actin Smooth Muscle antigen (SMA) for muscolarized unpaired arteries. Immunostaining for cytokeratins CK7/19 depicts the ductular reaction, which takes place around nonmalignant nodules: absence of staining could help to identify stromal invasion of well-differentiated malignant hepatocytes into portal tracts. Finally, the overexpression of three specific immunomarkers (Glypican 3- GPC3; Heat Shock protein 70- HSP70; Glutamine synthetase – GS) has been recognized to selectively label small and early HCC as compared to non-malignant counterparts (Table 1) [15, 16]. In the appropriate clinico-pathological context, the finding of 2 unequivocal positive immunomarker (out of 3 among GPC3, HSP70, and GS) can detect early HCC with a sensitivity of 72% and a specificity of 100% [15]. Because of the wide spectrum of histologic appearance of HCC, the differential diagnosis between HCC and other tumors involving the liver can be challenging. Secondary carcinomas may be difficult to assign to their origin but an appropriate immunohistochemical panel (Hep Par 1, pCEA, AFP, CD10, CKs 7, 8/18, 19, 20) may be very helpful to distinguish between primary and metastatic tumors (Table 1) (Figures 1, 2 and 3)[15, 16, 17]

**Table 1a:** List of the most important diagnostic biomarkers in HCC. An * has been added after the most useful diagnostic markers in clinical practice

Biomarker	Category	Role	Tissue Expression	Notes	Ref.
CD34, SMA	Endothelial marker (CD34), Smooth muscle marker (SMA)	Diagnostic*	Overexpressed	Highlights neovascularization (muscolarized unpaired arteries) and capillarization of sinusoids in HCC	[13, 15, 16, 17]
CK 7/19	Low molecular weight cytokeratins	Diagnostic	Not expressed	Positive staining in portal ductular of bening lesions	[13, 15, 16, 17]
GPC3	Cell surface heparin sulphate proteoglycan	Diagnostic*	Overexpressed (sensitivity =77%, specificity =96%)	It may also be seen in regenerating hepatocytes in a chronic hepatitis setting	[15, 16]
HSP70	Stress protein implicated in cell-cycle progression, apoptosis and tumorigenesis	Diagnostic*	Overexpressed (sensitivity =78%, specificity =95%)	-	[15, 16]
GS	Catalyzes the synthesis of glutamine from glutamate and ammonia	Diagnostic	Overexpressed (sensitivity =50%)	GS overexpression is able to mirror ß-catenin mutation	[15, 16]
Hep Par 1	Monoclonal antibody against urea cycle enzyme located in mitochondria	Diagnostic *	Expressed in hepatocytes	Most sensitive and specific marker of hepatocellular differentiation	[15, 16, 17]
pCEA	Glycoprotein present in the glycocalix of fetal epithelial cells and in small amounts in normal adults cells.	Diagnostic	Bile canaliculi and ductal epithelium but not in hepatocytes	Diffuse cytoplasmic expression in most adenocarcinomas (>90%). In HCC, pCEA reveals a specific “chicken-wire fence” canalicular pattern	[15, 16, 17]
AFP	Oncofetal protein expressed mainly in fetal gut, liver and yolk sac.	Diagnostic	HCC and germ cell tumors. Normal livers do not express AFP.	Staining is focal and sensitivity is about 30%	[15, 16, 17]
CD10	zinc-dependent metallopeptidase	Diagnostic	Expressed in several normal tissues, (ex. liver, small intestine, and brain)	Shows a canalicular pattern (Figure 1C) similar to pCEA, with lower sensitivity (50%).	[15, 16, 17]
miR-18, p-miR-18, -224	MicroRNA, small noncoding RNAs that control gene expression at a post-transcriptional level	Diagnostic	Higher expression levels in HCC samples	-	[20, 21]
miR-199a, 199a*, 195, 200a, 125a	MicroRNA	Diagnostic	Lower expression levels in HCC samples	-	[20, 21]
MiR- 92, 20, 18	MicroRNA	Diagnostic	Expression levels were inversely related with HCC degree of differentiation	-	[20, 21]
CK7, 8/18, 19, 20	Low molecular weight cytokeratins	Diagnostic *	Normal and neoplastic hepatocytes express cytokeratins CK 8 and 18 and about 70% of HCC are negative for CK7, CK19, and CK20	-	[15, 16, 17]

**Table 1b:** List of the most important prognostic biomarkers in HCC

Biomarker	Category	Role	Tissue Expression	Notes	Ref.
CD31, CD34, VWF	Endothelium-specific markers	Prognostic	Vessels and endothelium allowing semiquantitative assessment of tumor neovascularization (microvessel density)	High CD34-positive microvessels count predicts intrahepatic recurrence, shortened disease-free, OS and is associated with invasion and metastasis	[22–26]
VEGF	vascular endothelium growth factor, regulates angiogenesis through a complex network of molecular interactions with 5 ligands (VEGF-A to VEGF-E) that bind VEGF receptors (VEGFRs)	Prognostic	High tissue expression is a negative prognostic factor	High tissue expression of VEGF as a predictor of early mortality (hazard ratio, 2.15; 95% confidence interval, 1.26-3.78) and recurrence (hazard ratio, 1.69; 95% confidence interval, 1.23-2.33) following resection	[27, 28]
Hif	Hypoxia-inducible transcription factors reprograms gene expression to enhance the of proangiogenic mediators production in response to hypoxia.	Prognostic	High tissue expression is a negative prognostic factor	Hif-1a immunopositivity is predictor of worse disease-free and OS	[29, 30]
MMP family	Matrix Metalloproteinase are a group of >20 zinc-endopeptidases whose primary function is to degrade the extracellular matrix.	Prognostic	High tissue expression is a negative prognostic factor	Increased MMP-2 and MMP-9 expression correlates with recurrence and OS after liver transplantation[39] and resection	[39–44]
Cyclins and Cyclin Dependent Kinases	Diﬀerential activation of cyclins and cyclin dependent kinases (CDK) determine the transition between subsequent phases of the cell cycle.	Prognostic	High tissue expression is a negative prognostic factor	Tissue overexpression of cyclin A, D1 but not E[45] has been reported as an adverse prognostic factor after curative resection of HCC	[45, 46]
p16, p18, 27, 57	CDK inhibitors	Prognostic	Loss of expression is a negative prognostic factor	-	[46–50]
Mib-1 (Ki-67) and PCNA	Immuno-histochemical detection of nuclear antigens such as Ki-67 and PCNA are validated to assess cell proliferation	Prognostic	Tumors with increased growth rate have an increased risk of recurrence and shorter survival times	-	[46]
p53	Tumor suppressor gene/protein	Prognostic	Contradicting results	p53 mutational status by sequence analysis identified that patients with p53 mutant tumors are characterized by poorer survival [54–57] whereas the quantification of p53 mRNA expression did not correlate with survival [58].	[51–58]

P21-activated kinase 5 (PAK5) represents the latest family member of P21-activated kinases (PAKs) with Ser/Thr kinase activity. Expression of PAK5 gene in 25 out 30 HCC tissues has been demonstrated to be highly elevated with respect to the surrounding paraneoplastic tissue, making this gene an interesting diagnostic/prognostic marker for HCC [18].

A recent meta-analysis identified HMGB1 (high mobility group box 1) as a potential biomarker for HCC diagnosis, as it is significantly overexpressed at mRNA and protein level in HCC tissue samples, when compared with normal liver [19].

In an attempt to use microRNAs (miRNAs) to create a molecular classification of HCC, Murakami et al analyzed miRNA expression profiles in 25 pairs of HCC and adjacent non-tumorous tissue [20]. They found that three miRNAs exhibited higher expression in the HCC samples, whereas five were down regulated and others were inversely correlated with the degree of HCC differentiation (Table 2). Classification of samples as HCC or normal, based on these data, provided an overall prediction accuracy of 97.8%. More recently, Toffanin et al proposed a miRNA-based classification of three subclasses of HCC, displaying either activation of the Wnt pathway or enrichment of interferon response–related genes or activation of insulin-like growth factor 1-Receptor and Akt pathways [21].

### Prognostic tissue markers

Tumor hypervascularity is a well-known pathologic feature of HCC, where sustained angiogenesis guides the tumor to obtain a predominantly arterial blood inflow. Immunohistochemical staining for endothelium-specific markers such as CD-31, CD-34, or von Willebrand Factor allows for a semiquantitative assessment of the so called microvessel density, with significative prognostic power (Table 1) [22–26]. High tissue expression of angiogenic factors (Vascular endothelial growth factor –VEGF-) and of their regulators (Hypoxia-inducible –Hif-) are related to reduced OS and recurrence free survival and to early recurrence after resection (Table 1) [27–30].

Epithelial–mesenchymal transition (EMT) is characterized by the loss of cell-to-cell adhesion and gaining of mesenchymal phenotypes and is governed by specific transcription factors such as Slug (SNAI2), Snail (SNAI1), vimentin and Twist through which transforming cells progressively lose key adhesion molecules including E-cadherin and acquire unrestrained cell motility and metastatic potential. In a study by Zhang et al, a significant correlation between the dual over-expression of HIF-1α and SNAI1 and the reduced disease-free survival and poor prognosis was demonstrated in a cohort of HCC patients [31]. Even more interestingly, EMT phenotype can be reversed by reoxygenation [31]. Snail, Twist, but not Slug have been shown to correlate with metastatic potential and poor prognosis in HCC [32, 33]. In keeping with the hypothesis that suppression of E-cadherin reduces cell-cell adhesion and increases invasive potential, tumors displaying reduced expression of E-cadherin, tend to have a more aggressive clinical course [33–36]. Consistently, upregulation of the mesenchymal markers Vimentin and N-cadherin, 2 genes that are positively regulated during EMT, is also associated with poor outcome [37, 38].

Negative prognostic roles have been elucidated for matrix metalloproteinase (MMPs) MMP-1, MMP-2, MMP-7, MMP-9 and MMP-12 [39–44].

Cell cycle progression is a tightly regulated process in which the diﬀerential activation of cyclins and cyclin dependent kinases (CDK) determines the transition between subsequent phases of the cell cycle. Tissue overexpression of cyclin A, D1 but not E [45–46] has been reported as an adverse prognostic factor after curative resection of HCC. A confirmation of the detrimental eﬀect of p16 and p18 loss [46] and of p27 [47] and p57 [48–50] dysregulation on patient prognosis comes from studies analyzing their immunohistochemical expression (Table 1). Immunohistochemical detection of nuclear antigens such as Mib-1 (Ki-67) and PCNA are validated and reliable methods of assessing cell proliferation. Despite some variation in the cut-oﬀs used to categorize high proliferating versus low-proliferating tumors, more than 15 independent studies have confirmed that tumors with increased growth rate have an increased risk of recurrence and shorter survival times [46]. p53 accumulates within the nucleus in response to DNA damage and activates transcriptional programs leading to cell cycle arrest and apoptosis. Inactivation of the p53 gene by loss of heterozygosity or point mutation is a molecular event involving up to 60% of the HCCs [51]. Contradicting results are present with the association between p53 nuclear immunolabeling and decreased OS and DFS shown by some studies but refuted by others [52–58]. At least 50% of the HCCs display an aberrant activation of the mammalian target of rapamycin (mTOR) pathway, a master regulator of proliferation and survival programs currently under intense scrutiny in HCC for its prognostic [59] and therapeutic implications [60]. Epithelial growth factor (EGF) is one of the best characterized mitogens in epithelial cancer cells, acting on a family of receptors, which include the EGF receptor (EGF-R or c-erbB-1), Her-2 (c-erbB-2), Her-3 (c-erbB-3), and Her-4 (c-erbB-4). A comprehensive analysis of erbB receptors family expression in 100 surgical samples showed that EGF-R, Her-3, and Her-4 were expressed in more than half of the analyzed specimens, whereas Her-2 expression was present in only 21% [61]. At a transcriptional level, EGF-R but not EGF mRNA levels were related to the progression and recurrence of HCC [62, 63]. The prognostic role of Her-2 overexpression remains uncertain as only fluorescence in situ hybridization, but not immunohistochemical-based analysis has been found to correlate with tumor relapse and postoperative survival time [64].

The differential expression of miRNAs in HCC cells indicates the potential value of miRNA detection in the prediction of HCC prognosis (Table 2) [65].

**Table 2a:** List of miRNAs with different roles in HCC

miRNAs	Tumor associated	Role	Expression in tissue	Notes	Ref.
miR-18, p-miR-18, -224	HCC	Diagnostic	Higher expression levels of these miRNAs in HCC samples compared with normal tissue	-	[20, 21]
miR-199a,-199a*,-195, 200a, -125a	HCC	Diagnostic	Lower expression levels of these miRNAs in HCC samples compared with normal tissue	-	[20, 21]
miR- 92, -20, -18	HCC	Diagnostic	Expression levels were inversely related with HCC degree of differentiation		[20, 21]
miR-99a, -124, -139, -145 and -199b	HCC	Prognostic		Downregulation of these miRNAs was significantly associated with poor prognosis, shorter disease-free survival and features of metastatic tumors including venous invasion, microsatellite formation, absence of tumor encapsulation and reduced differentiation	[65]
miR-222, -135a, -155, -182, -10b, -17-5p,-221 and -21	HCC	Prognostic		Up-regulation of these miRNAs correlated with poor prognosis, such as increased risk of tumor recurrence and shorter overall survival	[65]
miR-122	HCC	Predictive of response to therapy	-	Restoration of miR-122 in HCC cells makes them sensitive to adriamycin and vincristine through down-regulation of Multidrug resistance (MDR)- related genes, the antiapoptotic gene Bcl-w and cyclin B1; it is also able to sensitize HCC cells to sorafenib	[76]
miR-122, -199a-3p	HCC	Predictive of response to therapy		These miRNAs affect sensitivity of HCC cells to doxorubicin	[76]
miR-21/anti-miR-21	HCC	Predictive of response to therapy		HCC cells transfected with pre–miR-21 were resistant to interferon-a (IFN-a)/5-FU, while cells expressing anti–miR-21 became sensitive to IFN-a/5- FU. Moreover, miR-21 expression in surgical HCC specimens was associated with the clinical response to the IFN-a/5-FU combination therapy and survival rate	[76]
miR-146a	HCC	Predictive of response to therapy		Induces resistance to interferon treatment through its ability to down-regulate SMAD4	[76]
miR-26	HCC	Predictive of response to therapy		Its low expression increases patients’ response to interferon therapy	[76]
miR-1274a	HCC	Predictive of response to therapy		Sorafenib was found to alter the expression of 14 miRNAs; among these miRNAs is miR-1274a, which is up-regulated by sorafenib resulting in repression of ADAM9, a protease involved in sorafenib targeted therapy	[76]

**Table 2b:** List of miRNAs with different roles in CCA

miRNAs	Tumor associated	Role	Expression in tissue	Notes	Ref.
miR-22, -125a, -127, -199a, -214, -376a, -424	CCA	Diagnostic	These miRNAs are down-regulated in intrahepatic cholangiocarcinoma (ICC) cells	These miRNAs could be served as potential diagnostic biomarkers for ICC	[194]
miR-21, -142-3p, -25, -15a, -193, -17-5p, -374, -106a, -224, -130b, -19a, -331, -324-5p, -20, 17-3p, -223, -15b, -103	CCA	Diagnostic	These miRNAs are up-regulated in ICC when compared with normal cholangiocytes	-	[195]
miR-98, -204, -338, -198, -302d, -328, -337, -302b, -184, -320, -371, -185, -222, -214, -373, -145, -200c, let-7a, let-7b, -197	CCA	Diagnostic	These miRNAs are down-regulated in ICC when compared with normal cholangiocytes	-	[195]
miR-21, -135b, -122, -27a, -29a, -429, -24, -203, -106b, -29b, -20a/-20b, -93, -30e, -30b, -151-3p, -10a, -181a, -96, -663b, -103, -221, -22, -107, -424, -340	CCA	Diagnostic	These miRNAs are up-regulated in CCA when compared with normal cholangiocytes	-	[196]
miR-451, -145, -99a, -125b, -630, let-7c, -144, -100, -127-3p, -139-5p, -337-3p, -1, -126, -376c, -517c+-519a, -520e	CCA	Diagnostic	These miRNAs are down-regulated in CCA when compared with normal cholangiocytes		[196]
miR-30c, -96, -30b, -100, -145, -125b, -127-3p	CCA	Diagnostic	miR-30c, -96, -30b, are up-regulated in CCA compared with pancreatic adenocarcinoma (PC); miR-100, -145, -125b, -127-3p are down-regulated in CCA compared (PC)	These specific miRNAs could be of aid in the differential diagnosis between these two neoplasms	[196]
miR-192, -675-5p, -652-3p, -338-3p, -126, -21	CCA	Prognostic		Decreased OS has been significantly associated with up-regulation of these miRNAs	[197–201]
miR-151-3p, -373	CCA	Prognostic		Decreased OS has been significantly associated with down-regulation of these miRNAs	[197–201]
miR-192, -21,-214	CCA	Prognostic		Nodal metastases have been found more frequently in patients with up-regulation of these miRNAs	[197, 200, 201]
Let-7g and miR-181b	CCA	Predictive of response to therapy		These miRNAs can alter the response to 5-fluorouracil-based antimetabolite S-1 and miRNAs of the Let-7 family can induce radio-sensibility	[219]
miR-21 and miR-200b	CCA	Predictive of response to therapy		These miRNAs can increase chemo-resistance to gemcitabine by interacting PTEN and PTPN12 respectively in CCA cell lines	[220]
miR-29b,-205, -221	CCA	Predictive of response to therapy		Downregulation of these miRNAs also present chemo-resistance to gemcitabine in CCA. Furthermore, PIK3R1 is identified as the common target of miR-29b and miR-221	[221]

### Predictive tissue markers

Selective inhibition of the VEGF pathway has been at the focus of intense research in the area of molecularly targeted therapy of HCC, particularly after sorafenib, a dual Raf/Map kinase inhibitor with concurrent inhibitory power on VEGF-R2, 3 and platelet-derived growth factor receptor, has become the standard of care in the treatment of advanced HCC [1, 66]. Because sorafenib is the only approved drug for advanced HCC in many countries, but exhibits a relatively modest activity, biomarkers to predict sorafenib efficacy could assist in identifying the minority of patients who are likely to benefit from the treatment [66]. Despite this clinical need, a lack of progress in this field occurred due to the scarcity of HCC tissues, particularly those obtained at advanced stages and before sorafenib treatment. In a recent analysis, MET (the receptor tyrosine kinase encoded by the homonymous MNNG-HOS transforming gene) showed a significantly higher expression after than before sorafenib treatment [27]. This is in line with literature suggesting that high MET expression correlates with hypoxia, resistance to anti-angiogenic therapies, and poor prognosis in HCC, and further supports the prognostic role of MET. In addition, tumor MET was found to be predictive of outcome in tivantinib-treated patients suggesting that tivantinib offsets the negative impact of MET, making survival of the MET-High treated patients similar to the MET-Low population [27].

Several studies have examined the downstream signaling molecules of Raf, such as ERK, in relation to response to sorafenib therapy. In a phase II study of sorafenib for advanced HCC, Abou-Alfa et al showed that patients with tumors expressing high phospho-(p-) ERK had a longer TTP [67]. However, recent studies have reported conflicting results [68, 69]; Arao et al [70] were the first to identify FGF3/FGF4 amplification in a patient with metastatic HCC who dramatically responded to sorafenib. Located at 11q13, the FGF3/FGF4 site is beside FGF19 and CCND1, 2 genes frequently reported to exert amplifications in HCC tissues [71–75]. According to the literature, FGF3/FGF4 amplification can be detected in 0% to 7% of HCC patients [73–75]. Among 10 other sorafenib responders, 3 patients also exhibited FGF3/FGF4 amplification. By contrast, no FGF3/FGF4 amplification was found in 38 patients who exhibited stable or progressive disease [73–75]. However, no validation studies have been performed and how FGF3/FGF4 amplification is associated with sorafenib efficacy remains unclear. Works performed in many types of cancer, including HCC, have shown that miRNAs can influence the sensitivity of tumors to therapy and that their expression can be altered by treatment (Table 2) [76].

## SERUM MARKERS

Serum biomarkers are striking potential tools for surveillance and early diagnosis of HCC thanks to the non-invasive, objective, and reproducible assessments they potentially enable [77]. Alpha-fetoprotein (AFP) testing and Ultrasonography (US) are the most widely used methods of HCC surveillance [78–82], representing the most cost-effective strategy. Data have indicated that AFP testing and US every 6 mo. affect disease-specific mortality compared to no intervention [odds ratio: 0.57, 95% confidence interval (CI): 0.37-0.89] [83]. However, there is increasing debate regarding the utility of AFP as a surveillance test [84–86]. Advances in technology and an increased understanding of HCC biology have led to the discovery of novel biomarkers (Table 3). To date, many biomarkers have been proposed as a complement or substitute for AFP in the diagnosis of HCC [77]. In particular, while a single marker alone could be poorly specific to predict this disease, using more than one marker at a time should greatly reduce the chance of errors from false-negative results.

**Table 3:** Serum markers in HCC

Marker	Function	Specificity	Sensitivity	Correlations	Notes
Alpha-fetoprotein (AFP)	Glycoprotein, synthetized during the early stages of fetal liver development. The biological function of AFP is still not well identified.	76%–94%	39-65%	Hepatocyte regeneration, during hepatocarcinogenesis and embryonic carcinomas	AFP sensitivity and specificity depend on the cut-off value chosen and on population characteristics
Lens culinaris Agglutinin (LCA) fraction of AFP	AFP glycoform, characterized by an elevated affinity to lectins such as Lens culinaris agglutinin	>95%	51%	Aggressiveness, poor differentiation, ki-67, distant metastasis	Sensitivity strictly depends on HCC diameter
Des-γ-carboxy prothrombin (DCP)	Abnormal prothrombin derived by an acquired defect in the post-translational carboxylation of the prothrombin precursor in HCC cells	48-62%	81-98%	Poor prognosis, portal vein invasion, intrahepatic metastasis, hepatic vein thrombosis, and capsular infiltration	A moderate diagnostic accuracy in HCC has been showed
Glypican-3 (GPC-3)	Heparan-sulphate proteoglycans. In HCC tissue, it promotes cell growth by stimulating Wnt signaling	87%-90%	53%-59%		GPC3 would serve also as a target for therapeutic intervention in HCC
P-aPKC-i	Serine-threonine kinases (PKC), important for apico-basal maintenance and cellular junction formation			Pathological differentiation, tumor size, invasion, and metastasis	Studied as tissutal marker more than serum marker
E-Cadherin	Transmembrane glycoprotein associated with inhibition of the formation of tight junctions among tumoral cells			Development of metastasis; poor tumoral differentiation	Studied as tissutal marker more than serum marker
b-Catenin	Cytoskeleton protein			Invasiveness and tendency to metastatization	Studied as tissutal marker more than serum marker
Human Carbonyl Reductase 2	Cytosolic enzyme involved in detoxification of the compounds derived from oxidative stress				Studied as tissutal marker more than serum marker
Vascular Endothelial Growth Factor	Role in angiogenesis, stimulating the proliferation and migration of endothelial cells and increasing vascular permeability	85%	65%	Portal vein emboli, poorly encapsulated tumors, microscopic vein invasion, and recurrence in HCC patients. Predictor of tumor aggressiveness, DFS, and OS in patients who underwent HCC resection	Associated with poor outcomes in patients treated with sorafenib, indicating that VEGF could be used as an indicator of clinical efficacy
Squamous Cell Carcinoma Antigen (SCCA)	Serin protease inhibitors	48.9%	84.2%	Tumor size	
a-l-fucosidase (AFU)	Lysosomal enzyme involved in degradation of fuco-glycoconjugates			PFS, OS, macrovascular invasion	
Transforming Growth Factor b1	Protein involved in inhibition of cell proliferation and triggering apoptosis			Hepatocarcinogenesis and tumor angiogenesis, tumor size, postoperative DFS and OS	
Embryonic Liver Fodrin (ELF)	Adaptor protein involved in TGF-β1 signaling pathway			HBsAg, tumor size, TNM and recurrence, postoperative DFS and OS	
Golgi protein-73	Golgi glycoprotein expressed in epithelial human cells	75%	69%		GP73 accuracy was higher than AFP, even if is less suitable for discriminating between primary malignant and benign tumors of the liver
Serum Anti-p53	Antibody directed against p53	91.52%	84.63%	AFP, tumor size, MELD and Child-Pugh score	P53 mutations are correlated with poorly differentiated cancer and shorter survival of patients with HCC
Chromogranin A (CgA)	Acidic glycoprotein contained in secretory granules of neuroendocrine cells			Degree of neuroendocrine differentiation of HCC	
Hepatocyte Growth Factor	Cytokine with wide ranges of effects; it stimulates hepatocyte proliferation including HCC cells through expression of its receptor, the c-met receptor			Poor survival	
Nervous Growth Factor	Cytokine involved in cancer growth, invasion and metastatization, in addition to its role in differentiation and survival of neuronal cells				The mechanism of NGF involvement in liver tissue remodeling processes and HCC remains unclear

### Alpha-fetoprotein (AFP)

Alpha-fetoprotein is a glycoprotein (MW 70 kDa), physiologically synthetized during the early stages of fetal liver development by the endodermal cells of the visceral yolk sac. The AFP expression by hepatocytes and endodermal cells of the yolk sac reduces after birth. The elevation of AFP can occur in hepatocyte regeneration, during hepatocarcinogenesis, and embryonic carcinomas [87]. The biological function of AFP is still not well identified. Since AFP is similar to albumin, it is possible that AFP function as a carrier for several ligands such as bilirubin, fatty acids, steroids, heavy metals, flavonoids, phytoestrogens, dioxin, and various drugs [88]. AFP evaluation is useful: a) for screening and diagnosis of HCC in patients at risk of developing HCC, in association to hepatic ultrasonography; (b) during staging within CLIP (Cancer of the liver Italian program) staging system; (c) as a marker for detecting tumor progression in patients with AFP-producing HCC. Analysis of recent studies has indicated that AFP testing lacks adequate sensitivity and specificity for effective surveillance [89–91]. AFP levels are normal in up to 40% of patients with HCC, particularly during the early stage of the disease (low sensitivity) [92–94]. Elevated AFP levels may be seen in patients with cirrhosis or exacerbation of chronic hepatitis or cholangiocarcinoma (low specificity) [95, 96]. In addition, some studies have indicated that AFP has substantially limited diagnostic accuracy in detecting small HCC [97]. The increase of AFP levels > 500 ng/ml is correlated with the tumor size: 80% of small HCC show no increase of AFP concentration. Furthermore, sensitivity of AFP decreases from 52% to 25% when tumor diameter is >3 cm and <3 cm, respectively [98]. Some patients with cirrhosis and/or hepatic inflammation can have an elevated AFP without the presence of tumor. The measurement of AFP serum concentration during the follow-up of patients after treatment is a helpful test in conjunction with computed tomography or magnetic resonance imaging [99]. A decrease of AFP levels less than 10 ng/ml within 30 days after treatment, indicates a favorable response to treatment [100]. However, the evaluation of serum AFP concentration is clinically significant when AFP is elevated before the therapy.

### Lens Culinaris Agglutinin Reactive AFP

There are several AFP glycoforms that differ in the binding affinity to lectins such as Lens culinaris agglutinin (LCA). Lens culinaris agglutinin reactive AFP (AFPL3%) or Lens culinaris agglutinin reactive fraction of AFP, is characterized by an elevated affinity to LCA and has been described as a more specific marker for HCC, because of its exclusive origin from cancer cells [101, 102]. Actually, the clinical utility of AFP-L3% and the ratio of AFP-L3% to total AFP remain unclear. The cut-off for AFP-L3% is set up >10% of total serum AFP. AFP-L3% measurement for HCC has a specificity >95% and a sensitivity of approximately 51% [103, 104]. In particular, its sensitivity ranges from 35–45% for small HCC (diameter<2 cm) to 80–90%, for HCC >5 cm [104]. The clinical utility of highly sensitive AFP-L3 in early prediction of HCC developing in patients with chronic HBV or HCV infection was recently evaluated in a large Japanese study, and results indicated that elevated AFP-L3 was an early predictor of HCC development even if AFP levels were low and suspicious US findings were absent. Elevated AFP-L3 was noted in 34.3% of patients 1 year prior to diagnosis of HCC [105]. Since AFP-L3%-positive patients develop early vascular invasion and intrahepatic metastasis, AFP-L3% is considered as a marker for the aggressiveness of HCC. In fact, AFP-L3% expression is related to progression from moderately differentiated to poorly differentiated tumors and seems to be connected with increased nuclear expression of Ki67 and with decreased expression of a-catenin, which is associated with distant metastasis [106, 107]. Beyond its utility as a prognostic factor, it could be used for patients’ follow-up after initial treatment. In particular, AFP-L3% expression after therapy is related to a shorter survival [108–110].

### Des-γ -Carboxy Prothrombin

Des-γ-carboxy prothrombin (DCP) or prothrombin induced by vitamin K absence (PIVKA) is an abnormal prothrombin derived by an acquired defect in the post-translational carboxylation of the prothrombin precursor in HCC cells [111]. DCP measurement for HCC has a sensitivity of 48–62% and a specificity of 81–98% [112]. DCP is a more specific HCC marker than AFP because it seems not to increase in other liver diseases. The accuracy of DCP is decreased in prolonged obstructive jaundice, intrahepatic cholestasis with vitamin k deficiency, and intake of warfarin. Higher DCP levels in HCC patients are associated with a poorer prognosis [113]. DCP is involved in tumoral angiogenesis, increasing genic expression of angiogenic factors such as EGF-R, VEGF, and MMP-2 and helping the proliferation and migration of human vascular endothelial cells [114]. DCP-positive patients frequently develop portal vein invasion, intrahepatic metastasis, hepatic vein thrombosis, and capsular infiltration [115–117]. In a recent article, Hakado et al. suggested that the elevation of AFP and DCP levels at 24 wk after the completion of IFN and ribavirin therapy were strongly associated with the incidence of HCC irrespective of virological response among Japanese patients with cirrhosis [118]. Recently, a meta-analysis indicated that DCP had moderate diagnostic accuracy in HCC. Further studies with rigorous design, large sample size and multiregional cooperation are needed in the future [119].

### Glypican-3

Glypican-3 (GPC3) is one of the members of heparan-sulphate proteoglycans [120]. Thanks to its binding to the cell membrane through the glyco-phosphatidylinositol anchors, it interacts with several growth factors, such as HGF and VEGF, contributing to the development of hepatic cancer [121]. Recent studies have shown that GPC3 levels are increased in HCC patients. GPC3 is able to differentiate between malignant and benign hepatic lesions; in fact, GPC3 levels are undetectable in healthy subjects and in benign hepatic disease patients (such as dysplastic or cirrhotic nodules) [122, 123]. In addition to its role of useful molecular marker for HCC diagnosis, GPC3 would serve also as a target for therapeutic intervention in HCC. Indeed, some immunotherapy protocols targeting GPC3 are under investigations; those include humanized anti-GPC3 cytotoxic antibody, peptide vaccine and immunotoxin therapies [124]. One of these is a phase II trial of codrituzumab (humanized monoclonal antibody against GPC3) in previously treated patients with advanced hepatocellular carcinoma. Unlucky, in this clinical trial, codrituzumab was not found be effective against liver cancer. It was suggested though that a higher dose of codrituzumab or selecting patients with high level of glypican-3 or its mediator CD16 might improve outcome [125].

### P-aPKC-i, E-Cadherin, b-Catenin

P-aPKC-i, E-cadherin, and b-catenin play an important role in tight-junctions formation among tumor cells. P-aPKC-i is a member of the family of serine-threonine kinases (PKC) that play an important role in cellular proliferation and differentiation [126]. P-aPKC-i is very important for apicobasal maintenance and cellular junction formation [127]. In normal liver tissue, it is localized at the apical membrane, while in HCC it is localized at the basal membrane and in cytoplasm. Probably, the high expression of aPKc-i causes the loss of cell polarity and cellular junction, leading to metastasis [128]. E-cadherin is a transmembrane glycoprotein, connected with its intracellular domain, through b-catenin and other catenins, to the acting cytoskeleton. The reduced expression of E-cadherin is associated with inhibition of the formation of a tight junction among tumoral cells, and is correlated to development of metastasis and a poor tumoral differentiation. B-catenin overexpression in HCC tissues seems to be involved in activation of the WNT signaling pathway and in expression of c-myc, cyclin D, VEGF, and other genes related to cell proliferation [129, 130].

### Human Carbonyl Reductase 2

Human carbonyl reductase 2 (HCR2) gene encodes a cytosolic enzyme that is expressed in the human liver and kidney and is involved in detoxification of the compounds derived from oxidative stress. Different studies showed that a decreased expression of HCR2 in HCC tissues contributes to cancer growth because it increases the cellular damage induced by ROS and other carcinogens [131].

### Vascular Endothelial Growth Factor

The development of solid tumors is strictly correlated with angiogenesis. Vascular endothelial growth factor (VEGF) plays an important role in angiogenesis, stimulating the proliferation and migration of endothelial cells and increasing vascular permeability. VEGF levels are higher in HCC patients than in chronic hepatic disease patients, and in advanced HCC compared to early HCC [132, 133]. Vascular damage and invasion by cancer cells are fundamental for distant metastasis. Platelets, activated by vascular invasion of HCC cells, release VEGF. VEGF is considered a possible tumor marker for HCC metastasis. High serum VEGF is associated with portal vein emboli, poorly encapsulated tumors, microscopic vein invasion, and recurrence in HCC patients. VEGF is a predictor of tumor aggressiveness, disease-free survival, and overall survival in patients who underwent HCC resection [134, 135]. There are studies showing that single nucleotide polymorphisms (SNPs) in VEGF are a predictive factor of survival in patients with HCC resection [136]. Based on these studies, it is speculated that SNPs in genes related to angiogenesis, including VEGF, may affect tumor progression and recurrence of the disease in patients after transplantation [137]. The presence of elevated serum VEGF levels and recurrence of HCC in patients after liver transplantation seems to be closely associated with poor prognosis [138]. High level of VEGF is associated with poor outcomes in HCC patients treated with sorafenib, indicating that VEGF could be used as an indicator of clinical efficacy in patients with HCC. However, better designed studies are needed to strengthen our findings [139]. An interesting work has shown that VEGF-siRNA enhanced the chemosensitivity of doxorubicin in Hep3B cells at least in part by suppressing the expression of anti-apoptotic genes. Therefore, the downregulation of VEGF by siRNA combined with doxorubicin treatment has been shown to yield promising results for eradicating HCC cells [140]. Moreover, another article has described that serum VEGF level in liver cancer patients can be used as a prognostic indicator for evaluating the efficacy of RFA treatments [141].

### Squamous Cell Carcinoma Antigen (SCCA)

Squamous Cell Carcinoma Antigen (SCCA) belongs to family of serpins, serin protease inhibitors [142]. SCCA expression, as well as AFP production, could be the consequence of the dedifferentiation often observed in HCC. HCC patients show higher SCCA serum levels than cirrhotic patients do. There is no clear correlation between SCCA expression in tissues and SCCA serological levels. SCCA may be used for HCC diagnosis, since it shows a sensitivity of 84.2% and a specificity of 48.9%. Given that SCCA is inversely correlated with tumor size, it is helpful for early HCC diagnosis and in screening of chronic hepatic disease patients [143]. A recent meta-analysis indicated that SCCA and SCCA-IgM exhibit moderate diagnostic accuracy as novel tumor makers of HCC, although the value of the combination of SCCA/SCCA-IgM and AFP requires further investigation [144].

### a-l-Fucosidase

a-l-fucosidase (AFU) is a lysosomal enzyme found in all mammalian cells, whose role consists in the degradation of fuco-glycoconjugates [145]. Its activity is higher in HCC patients than in healthy individuals and in chronic hepatic disease patients. AFU measurement is useful in association with AFP in the early diagnosis of HCC. Moreover, here is a positive correlation between AFU levels and tumor size in HCC patients [145–149]. A large-scale, long-term study, using a cut-off of preoperative AFU>35 U/l, showed that preoperative AFU was an independent prognostic factor of overall survival (OS) (P=0.008; hazard ratio: 2.333; 95% confidence interval: 1.249–4.369). Patients with a preoperative AFU>35 U/L had a lower recurrence-free survival rate and an OS rate than those with AFU⩽35 U/L, and they have a higher tendency to form macrovascular invasion. Furthermore, the prognostic significance of AFU>35 U/L could also be applied to patients with alpha-fetoprotein levels of ⩽400 ng/ml [150].

### Transforming Growth Factor b1 and Embryonic Liver Fodrin (ELF)

Transforming growth factor b1 (TGF-b1) arrests the cell cycle in the G1 phase, inducing inhibition of cell proliferation and triggering apoptosis. In normal liver tissues, TGF-b1 is produced only by nonparenchymal cells (Kupffer cells, storing cells, and endothelial cells). Recent studies showed that TGF-b1 serum levels are increased in HCC patients and are correlated with hepatocarcinogenesis and tumor angiogenesis [151–153]. The levels of TGF-b1 mRNA are higher in larger tumors. HCC cells show resistance to TGF-b1 growth inhibition because in tumoral cells there is an overexpression of cyclin D1 correlated with the dysregulation of the cell cycle and tumor progression [154–156]. TGF-β1 signaling pathway requires an adaptor protein, Embryonic Liver Fodrin (ELF). The prognostic significance of ELF for hepatocellular carcinoma (HCC) has not been clarified. While the expression of TGF-β1 in HCC tissues was significantly higher than that in normal liver tissues, the expression of ELF in HCC tissues declined markedly. ELF protein was correlated with HBsAg, tumor size, tumor number, TNM and recurrence. Data also indicated a significant negative correlation between ELF and TGF-β1. Patients with high TGF-β1 expression or/and low ELF expression appeared to have a poor postoperative disease-free survival and overall survival compared with those with low TGF-β1 expression or/and high ELF expression. Furthermore, the predictive range of ELF combined with TGF-β1 was more sensitive than that of either one alone [157].

### Golgi protein-73

Golgi protein-73 (GP73) is a Golgi glycoprotein expressed in epithelial human cells [158]. Physiologically, GP73 is expressed in biliary epithelial cells but not in hepatocytes. In liver disease, GP73 expression is increased in hepatic cells [159]. GP73 values are higher in early HCC patients than in cirrhotic patients. GP73 is considered a possible marker for HCC; in fact, it shows a specificity of 75% and a sensitivity of 69%. There are several isoforms of GP73 correlated with different levels of glycosylation [160]. Therefore, some isoforms are more specific for HCC. Further studies are needed to confirm the role of GP73 in HCC diagnosis. An interesting meta-analysis showed that in HCC diagnosis, the accuracy of GP73 was higher than that of AFP, and that GP73 + AFP exhibited significantly higher diagnostic accuracy than did GP73 or AFP alone[161]. Although the literature suggests that GP73 is a valuable serum marker in patients with HCC, the serum concentration may also be increased in patients with solid benign liver tumors. Therefore, a GP73 assay is less suitable for discriminating between primary malignant and benign tumors of the liver [162].

### Serum Anti-p53

The p53 gene is an onco-suppressor gene encoding a nuclear phosphoprotein (p53 protein) that inhibits cellular proliferation and transformation [163]. Mutations of the p53 gene have been reported in several human cancers. P53 alterations are not an early event in HCC, but occur at the late stages of hepatocarcinogenesis and are detected in 30–50% of HCC patients. P53 abnormalities are connected with the prognosis and survival of HCC patients. P53 mutations are correlated with poorly differentiated cancer and shorter survival of patients with HCC [164–166]. An interesting study reported that sensitivity, specificity, PPV, NPV and accuracy test of anti P53 antibody positive patients are 91.52%, 84.63%, 90.34%, 80.2% and 74.8% respectively[167]. It correlates positively with AFP, tumor size and staging, MELD score and Child-Pugh score. Non-B non-C HCC showed high serum prevalence of anti-p53 as viral-associated HCC suggesting an evidence of high onchogenecity. It appears of much benefit in diagnosis, follow up and differentiation from cirrhosis in presence of low levels of alpha-fetoprotein [167].

### Chromogranin A

Chromogranin A (CgA) is an acidic glycoprotein contained in secretory granules of neuroendocrine cells [168]. Different studies show high serum Cg-A concentrations in patients with HCC, suggesting that CgA might represent a useful marker in monitoring cirrhosis patients for the early detection of HCC. Moreover, Cg-A levels seems to be linked to the degree of neuroendocrine differentiation of HCC [169, 170]. Since CA levels increase in both HCC patients and in cirrhotic patients, it shows a low diagnostic specificity. Patients with a higher CgA serum concentration show a poorer outcome than those with lower CgA levels [171]. Additionally, CgA can be utilized in monitoring the efficacy of HCC treatment.

### Hepatocyte Growth Factor

Hepatocyte growth factor (HGF) is a cytokine having a wide range of effects, from embryonic development and liver regeneration to protection and/or repair of various organs, including kidney, lung, and cardiovascular system [172, 173]. HGF stimulates hepatocyte proliferation including HCC cells through expression of its receptor, the c-met receptor. HGF can be detected in the serum of hepatic chronic disease patients. The increase of HGF serum levels in cirrhotic patients is an indicator of HCC development [174, 175]. HGF serum levels higher than or equal to 1.0 ng/ml have been correlated with poor survival. Therefore, pre-operative high HGF levels are related to development of post-operative complications, such as liver failure [176]. HGF can be helpful in assessing hepatic function before surgery and for predicting a patient's prognosis. Moreover, elevated HGF serum levels, after surgery, is able to predict early tumor recurrence and metastasis [177].

### Nervous Growth Factor

Nervous growth factor (NGF) is involved in cancer growth, invasion and metastatization, in addition to its role in differentiation and survival of neuronal cells [178]. Various studies show that NGF is over-expressed in approximately 60% of human HCC tissues compared to the surrounding liver tissue with cirrhosis and chronic hepatitis, suggesting a role in HCC progression [179–181]. In fact, hepatic stellate cells express neurotrophins and their receptors are increased during hepatic regeneration [182, 183]. NGF and its related receptors play an important role in modulating the physiopathology of the intrahepatic biliary epithelium in the course of liver tissue remodeling processes and HCC progression. The mechanism of NGF involvement in liver tissue remodeling processes and HCC remains unclear.

## CHOLANGIOCARCINOMA

## INTRODUCTION

Cholangiocarcinoma (CCA) arises from the neoplastic proliferation of cholangiocytes, the epithelial cells in the biliary tree. It is an aggressive malignancy, characterized by early lymph node involvement and distant metastasis, with 5-year survival rates of 5%-10% [184]. The identification of new biomarkers with diagnostic, prognostic or predictive value is especially important as resection (by surgery or combined with a liver transplant) has shown promising results and novel therapies are emerging [185]. However, the relatively low incidence of CCA, high frequency of co-existing cholestasis or cholangitis (primary sclerosing cholangitis –PSC- above all), and difficulties with obtaining adequate samples, despite advances in sampling techniques and in endoscopic visualization of the bile ducts, have complicated the search for accurate biomarkers. As the clinical presentation of CCA can mimic benign dominant biliary strictures, the major challenge lies in identifying potential biomarkers that detect early dysplasia and CCA.

The goal of theranostic markers is to predict the response to a specific therapy. Current standardized regimens available for medical therapy in CCA have limited effectiveness and considerable side effects. Therefore, there is a crucial need for development of targeted therapies that interfere with growth and progression of cancer cells, sparing normal cells.

## TISSUE MARKERS

### Diagnostic tissue markers

Microscopically, some CCA grow in a cord-like pattern reminiscent of the trabeculae of HCC. The cords are always separated by a connective tissue stroma rather than by sinusoids; canaliculi and bile are also absent (Figure 1D). Immunohistochemistry could be of aid in the differential diagnosis, as almost all CCA are diffusely positive for cytokeratin 7 and 19 (Figure 1E and 1F, respectively), whereas only a few cases of HCC are positive. The hepatocyte antigen (Hep Par 1) is expressed by HCC but not by CCA.

As up to a quarter of patients undergo unnecessary surgical resection for suspected CCA-related strictures, which turn out have benign etiology[186], it is crucial to identify a highly sensitive test (beyond cytology or histology) that may reduce the number of unrequired surgeries. Assessment of polysomy by Fluorescent In Situ Hybridization (FISH) has shown the greatest accuracy in brush cytology specimens: some studies have found that the inclusion of the 9p21/p16 deletion in FISH analysis of indeterminate strictures increased the sensitivity of FISH-polysomy for pancreatobiliary tract cancers from 58% to 89% and from 70% to 76% [187–190]. The importance of sampling the biliary tree at multiple locations, regardless of the location of the dominant stricture, was demonstrated in a recent study that found that multifocal polysomy analysed from multiple sites carried a greater risk of CCA diagnosis than polysomy detected at a single location [191].

Kirsten rat sarcoma viral oncogene homolog (Kras) is a GTPase downstream of the EGFR receptor that activates proteins involved in cell growth and proliferation. The high specificity of Kras mutational analysis in biliary strictures can be useful, but the low sensitivity precludes it from diagnostic use as a unique biomarker. When used in combination with cytology, sensitivity increased to 100% [191].

Recently, the methylation status of 13 candidate genes was investigated in tissue samples and biliary brush samples of CCA and normal controls using quantitative methylation-specific PCR (polymerase chain reaction)[192]. The Authors have defined a novel biomarker panel (CDO1, CNRIP1, SEPT9, and VIM) that accurately identifies malignancy in biliary brush and tissue samples from CCA patients. The cancer specificity was also retained among PSC cases, which are characterized by the presence of inflammatory biliary epithelium. Combining the biomarker panel with conventional brush cytology/ biopsies increased the sensitivity for CCA detection [191]. This promising biomarker panel, if validated in larger series, could be employed for the development of a routine molecular test for monitoring PSC patients at high risk for CCA development.

miRNAs expression profiles, detected by qRT-PCR (quantitative real time polymerase chain reaction) and/or ISH (In Situ Hybridization), might be valuable tools for the diagnosis of biliary tree carcinomas (Table 2) [193–195].

### Prognostic tissue markers

Although conventional clinicopathologic features including histologic grade, pathologic stage, clinical stage and lymph node metastasis have become prognostic factors for predicting the clinical outcome of cancer patients, they have proven to be frequently inadequate to predict either the progression or the biological behavior of cancers accurately. Hence, new prognostic biomarkers, including miRNAs [196–200], that could reflect the biological behavior of CCA are currently under investigation (Table 2).

EGFR and KRAS mutations are not uncommon in biliary tract neoplasms. EGFR mutation detected on FFPE tissue samples of resected specimens for CCA was an independent prognostic parameter on multivariate analysis, along with tumor stage [201]. EGFR immunohistochemical overexpression on tumor tissue samples was significantly related to many clinicopathological features of CCA (macroscopic type, nodal metastases, lymphatic vessel invasion, perineural invasion and tumor stage) and was an independent prognostic factor related to decreased survival [202].

‘Cadherin switch’, a process in which cells shift to express different isoforms of the cadherin transmembrane protein and usually refers to a switch from the expression of E-cadherin to N-cadherin, is one aspect of EMT and can have a profound effect on tumour invasion/metastasis. Nitta et al. demonstrated that the expression of E-cadherin, N-cadherin and S100A4 was each an independent and a significant prognostic factor in extrahepatic cholangiocarcinoma (ECC) [203]. “Cadherin switch” also was independently associated with poor prognosis in patients with ECC [203].

### Predictive tissue markers

Several growth factor tyrosine kinases are implicated in carcinogenesis and progression of CCAs. These include the ERBB family of receptor tyrosine kinases, fibroblast growth factor receptor (FGFR), and the hepatocyte growth factor (HGF) receptor, MET.

The ERBB family of receptor tyrosine kinases is comprised of four different receptors, ERBB1 or EGFR, ERBB2 or HER-2/neu, and ERBB3 and 4 [204]. The EGFR activation leads to downstream activation of mitogen-activated protein kinase (MAPK), a well-known oncogenic signaling pathway. Over-expression of ERBB2, an EGFR family member, has been linked to biliary epithelial tumor formation in mice 43 and humans, with a reported prevalence of 3% [205]. Erlotinib, an EGFR inhibitor, has had limited success in human CCA clinical trials [206]: this may partly be due to an insufficient understanding of EGFR signaling in the molecular pathogenesis of CCA, and failure to select a patient population overexpressing EGFR. Indeed, the reported prevalence of EGFR mutations and amplification is only 15% and 5%, respectively [206]. A recent molecular and genomic characterization of 104 surgically resected CCAs demonstrated significant upregulation of HER2 signaling in tumors with the most malignant phenotype. The HER2 upregulation was associated with poor prognosis and frequent coactivation of ERBB3 and EGF [207]. Lapatinib, a dual inhibitor of EGFR and HER2, was significantly more effective in inhibition of CCA cell lines than trastuzumab, which selectively inhibits HER2 [207]. MET tyrosine kinase plays an integral role in carcinogenesis by promoting tumor invasion, protection from apoptosis, and angiogenesis; binding to HGF or scatter factor activates MET. HGF and MET expression is enhanced in CCAs, and is associated with activation of ERBB family members, especially ERBB2. An integrative genomic analysis of ICC identified a proliferation class of ICC that is characterized by activation of oncogenic signaling pathways such as MET [208]. MET amplification has been described in malignancies including gastric, esophageal, ovarian, and non-small cell lung cancer, and is associated with a poor clinical outcome [206]. MET amplification is also linked to resistance to EGFR and ERBB2 inhibitors and may predict sensitivity to MET inhibitors [206]. FGFR is a receptor tyrosine kinase involved in a myriad of biological processes including cell transformation, angiogenesis, and tissue repair [209]. Fusions of the FGFR gene have been reported in solid cancers. Recently, FGFR2- BICC1 gene fusion was described in two cases of CCA by Wu et al [210]. A high prevalence (13.6%) of FGFR2 gene fusions was reported in a cohort of 102 CCA cases [209]. In addition to detecting several cases with FGFR2-BICC1 fusions, a single case of a novel FGFR gene fusion, FGFR2-AHCYL1, was also identified in this cohort. Overexpression of FGFR2-BICC1 is associated with enhanced cell proliferation and altered cell morphology. Based on mechanistic studies, Wu et al proposed that FGFR fusion partners mediate oligomerizations, which initiates activation of the respective FGFR kinase in tumors harboring these mutations [210]. Only cells harboring the FGFR gene fusions were sensitive to FGFR inhibitors [209, 210], indicating a role for targeted FGFR kinase inhibition in patients with tumors containing these gene fusions.

Binding of growth factors, such as HGF and FGF, to their respective receptor tyrosine kinases leads to activation of the PI3K cell-signaling pathway. Subsequent AKT activation leads to phosphorylation and activation of the mTOR pathway [211]. Deregulation of the PI3K-AKT-mTOR pathway fosters tumor development, cell proliferation and survival, tumor invasion, and angiogenesis. PIK3CA mutations were identified in 5 of 94 resected CCA specimens using MassARRAY technology [211]. Exome sequencing of 32 CCAs detected somatic mutations in several members of the PI3K pathway [212]. Various PI3K pathway inhibitors are under investigation in multiple clinical trials of human cancer [206]. A phase I trial of everolimus, an mTOR inhibitor, in combination with gemcitabine and cisplatin demonstrated promising clinical activity in patients with treatment refractory solid tumors, including CCA [206]. Mutations in KRAS have been frequently described in CCA, along with other mutations in NRAS, BRAF, and downstream MAPK effector pathways. The proliferation subclass reported by Sia et al was notable for MAPK pathway activation [207]. Strategies to therapeutically target tumors with KRAS mutations have focused on targeting downstream effector pathways of KRAS such as Raf/MEK/ERK and PI3K/AKT.

A gain-of-function mutation in isocitrate dehydrogenase 1 (IDH1), leading to inhibition of α-ketoglutarate, has been seen in 23% of ICC cases and a minority (0%-7%) of ECC tumors [213–215]. In-vivo studies have suggested that drugs mimicking α-ketoglutarate alone or in combination with inhibitors of mutant IDH1 can reverse the increased histone methylation [215]. Additionally, IDH enzymes are stable therapeutic targets because the mutation appears early in oncogenesis and is maintained throughout progression to high-grade lesions [214].

ICC and ECC display differences in growth patterns, symptoms, treatment response, and survival and may therefore benefit from different therapeutic strategies. Wiggers et al performed a systematic review of the literature and meta-analysis including 4458 CCA patients, to analyze differences in the immunohistochemical profile of these distinct tumors [216]. 18 markers showed a statistically significant difference in expression between ICC and ECC and among these, 3 biomarkers included potential targets of therapy: EGFR, c-erbB-2 and VEGF-A (vascular endothelial growth factor-A) [216].

Although surgical resection with post-surgical adjuvant therapy such as chemotherapy and radiation therapy are used in treatment of biliary tract carcinomas, including CCA, the current 5-year overall survival for patients only attain 15% or less [217]. MiRNAs could act as either oncogenes or tumor-suppressor genes to participate in the regulation of multiple cancer cells functions by means of altering the expression of target genes. Thus, miRNAs can be considered theranostic markers as well as new potential therapeutic agents for cancers [218–220].

## SERUM MARKERS

Acquisition of tumor tissue for histology or biomarker testing can be difficult and requires more invasive procedures. A recent review by Viterbo et al well describe the actual scenario of diagnostic, prognostic and theranostic markers in CCA [221]. Table 4 summarizes the most important serum markers evaluated in CCA.

**Table 4:** Serum markers in CCA

	Markers	Specificity	Sensitivity	Notes
Diagnostic markers	CA19-9	67%-98%	38%-93%	
	CEA	50%-87.8%	33%-84%	
	Interleukin-6	low	100%	
	CYFRA 21-1 and MMP-7	low	Variable, depending on the cut-off value	Using CYFRA 21-1 and MMP-7 with CEA and CA19-9 demonstrated the highest diagnostic accuracy of 93.9%[
	SSP411			Elevated in the bile of CCA patients and recently found to successfully distinguish CCA from choledoco-lithiasis as a single serum-based biomarker
	miRNA			CCA-specific miRNA expression profiles has been identified: miR-125a, -31, and -95 are downregulated, while multiple miRNAs are upregulated as compared to nonmalignant cholangiocytes. Low specificity of single mi-RNA suggests that the most effective use of miRNAs is likely as multimarker panels specific for CCA
	CTC		17%-23% (Using a cut-off of 2 CTCs/7.5 mL of peripheral blood)	More useful as prognostic markers, correlating with poor overall survival
Prognostic markers	miRNA multi-marker panels			Correlation with overall survival and rate of metastasis
	EGFR			Over-expression of EGFR is prognostic of decreased overall survival
	CYFRA 21-1			values above 2.7-3 ng/mL is prognostic of decreased overall survival
Theranostic markers	EGFR mutations			In a phase II study of single agent erlotinib in patients with advanced biliary cancer, stable disease was reached in 17% of patients
	Upregulation of vascular endothelial growth factor (VEGF)			It is associated with an EGFR inhibitor-resistant phenotype. Vandetanib, a dual inhibitor of VEGF and EGFR, has shown prolonged time to metastasis in CCA tumors that harbor both mutations
	KRas/BRAF mutations			Several studies suggest the potential application for targeted therapy with vemurafenib in this population, but not with EGFR-inhibitor
	HER2 overexpression			This 4%-5% of CCA may benefit from targeted anti-HER2 therapy
	Expression of miRNAs			The increased expression of miRNAs predicts a favorable response to gemcitabine treatment. Moreover, treatment of cholangiocytes with miR-494, which is down-regulated in CCA, induced cell-cycle arrest in tumor cells while sparing normal cells

### Diagnostic markers

The most frequently used serologic markers of CCA are CA19-9 and CEA. Sensitivity/specificity of CEA and CA 19-9 are 33%-84%/50%-87.8% and 38%-93%/67%-98%, respectively [222–226]. Although CA 19-9 may have a role in the diagnostic algorithm, especially in patients with primary sclerosing cholangitis (PSC) in the absence of concurrent cholangitis or pancreatitis, the low accuracy of the test limits its role in screening and early diagnosis. Thus, novel biomarkers with potential diagnostic utility are needed. In malignant epithelial cells, activated proteases release cytokeratin-19 fragments (CYFRA 21-1) into the bloodstream [227]. Several studies have shown elevated CYFRA 21-1 expression in CCA, but sensitivity varied depending on the cut-off value [222, 227, 228]. The elevation of CYFRA 21-1 and matrix metalloproteinase-7 (MMP-7) in various malignancies reduce their specificity; preclude their use in clinical practice as diagnostic biomarkers. Thus, the solution can be represented by combinations of more serum markers to improve sensitivity without reducing specificity. Using CYFRA 21-1 and MMP-7 with CEA and CA19-9 demonstrated the highest diagnostic accuracy of 93.9% [228]. Interleukin-6 (IL-6), a growth factor for bile duct epithelium [229], has a sensitivity as high as 100% in diagnosing CCA [230], but a low specificity, considering its elevated levels in hepatocellular carcinoma, benign biliary disease, and metastatic lesions, limiting its specificity [231]. Spermspecific protein 411 (SSP411) is one such protein which is elevated in the bile of CCA patients and recently found to successfully distinguish CCA from choledocholithiasis as a single serum-based biomarker [232]. Several recent studies have evaluated the role of miRNAs in CCA. When miRNAs are dysregulated in cancers, they can be detected in blood in free form and can be used as potential diagnostic markers [233]. The utility of miRNAs lies in their tissue-specific patterns of expression. In particular, CCA-specific miRNA expression profiles has been identified (miR-125a, -31, and -95 are downregulated, while multiple miRNAs are upregulated as compared to nonmalignant cholangiocytes) [234, 235]. The most commonly overexpressed miRNA in CCA is miR-21[236–238], but its low specificity suggests that the most effective use of miRNAs is likely as multimarker panels specific for CCA. As for other cancers, CTCs in CCA were found to be prognostic of poor overall survival. Using a cut-off of 2 CTCs/7.5 mL of peripheral blood, the sensitivity of CTCs for CCA diagnosis is only 17%-23% [239, 240]. Despite their poor diagnostic utility, CTCs are potentially useful in detection and monitoring treatment of metastatic spread in clinical practice. In a recent study, Janvilisri et al attempt to identify serum tumor markers for CCA that can effectively distinguish CCA from benign biliary tract diseases (BBTDs), using a proteomic approach. In addition to identifying several proteins previously known to be differentially expressed in CCA and BBTDs, they also discovered a number of molecules that were previously not associated with CCA, such as FAM19A5, MAGED4B, KIAA0321, RBAK, and UPF3B[241]. Further validation of these proteins has the potential to provide a biomarker for differentiating CCA from BBTDs.

### Prognostic markers

Recent studies have been successful in establishing miRNA signatures that can discriminate between CCA and normal tissue as well as provide prognostic clues [234, 242]. As various miRNA expression patterns correlate with overall survival and rate of metastasis, the identification of accurate and predictive multi-marker panels can identify patients in need of more aggressive management earlier [221]. Over-expression of EGFR and CYFRA 21-1 values above 2.7-3 ng/mL [222, 228] were each prognostic of decreased overall survival [243, 244].

### Theranostic markers

The term “theranostic” was coined to define ongoing efforts in clinics to develop more specific, individualized therapies for various diseases, and to combine diagnostic and therapeutic capabilities into a single marker. EGFR mutations can be unique to CCA [245] or identical to those in non-small cell lung cancer [246]. A phase II study of single agent erlotinib in patients with advanced biliary cancer, stable disease was reached in 17% of patients [247]. Upregulation of vascular endothelial growth factor (VEGF) is associated with an EGFR inhibitor-resistant phenotype [248, 249]. Vandetanib, a dual inhibitor of VEGF and EGFR, has shown prolonged time to metastasis in CCA tumors that harbor both mutations [250]. Kras is one of the most frequently mutated genes in CCA, while BRAF mutations have been identified in up to 22% of CCAs [251, 252]. Several studies suggest the potential application for targeted therapy with vemurafenib in this population, but not with EGFR-inhibitors [251, 253]. An on-going phase II “basket” study of vemurafenib in non-melanoma solid tumors harboring BRAF mutations demonstrated stable disease at 8 wk in 4/7 CCA patients, partial response in 2/7 at 24 wk and the remaining 1/7 with disease progression (clinical trial # NCT01524978). The small minority (4%-5%) of CCA cases that overexpress erythroblastosis oncogene B2 (ErbB2 or HER2) [254] may benefit from targeted anti-HER2 therapy [255]. The increased expression of some miRNAs predicts a favorable response to gemcitabine treatment [234, 256]. The potential of miRNAs lies not only in their theranostic utility, but also as therapeutic agents. Treatment of cholangiocytes with miR-494, which is down-regulated in CCA, induced cell-cycle arrest in tumor cells while sparing normal cells [257, 258].

## CONCLUSIONS

Our review focused on CCA-specific tissue and serum biomarkers that may help in the early diagnosis of cancer or guide therapeutic decisions in the case of inoperable malignancy. A non-invasive serologic screening test with a high sensitivity or multi-marker panels could be very advantageous for patients and very useful in clinical practice. Despite the more invasive nature of tissue markers, high-risk patients would benefit from their high specificity. Further validations of novel biomarkers in degenerative/cancer liver disease [259] and multicenter international studies are needed.

### Core Tip

HCC represents the sixth most common cancer worldwide and the second leading cause of cancer-related death. Despite the high incidence, treatment options for advanced HCC remain limited and unsuccessful, resulting in a poor prognosis. Despite the major advances achieved in the diagnosis of HCC, only one third of the newly diagnosed patients are presently eligible for curative treatments. Cholangiocarcinoma (CCA) is an aggressive malignancy, characterized by a poor prognosis. The identification of new biomarkers with diagnostic, prognostic or predictive value is especially important as resection (by surgery or combined with a liver transplant) has shown promising results and novel therapies are emerging. Improving our knowledge about serum and tissutal markers could ultimately lead to an early diagnosis and better and early treatment strategies for this deadly disease. In this review, we attempt to analyze the existing literature on this argument.

## Abbreviations

HCC: hepatocarcinoma
HCR2: Human carbonyl reductase 2
HCV: hepatitis c virus
HGF: Hepatocyte growth factor
IFN: interferon
IL-6: Interleukin-6
LCA: Lens culinaris agglutinin
MMP-2: matrix metalloproteinase-2
MMP-7: matrix metalloproteinase-7
miRNA: micro RNA
NGF: Nervous growth factor
NPV: Negative Predictive Value
OS: overall survival
PIVKA: prothrombin induced by vitamin K absence
PPV: Positive Predictive Value
PSC: primary sclerosing cholangitis
RFA: radiofrequency ablation
SCCA: Squamous Cell Carcinoma Antigen
SNPs: single nucleotide polymorphisms
SSP411: Spermspecific protein 411
TGF b1: Transforming Growth Factor b1
VEGF: vascular endothelial growth factor
FFPE: formalin-fixed paraffin-embedded
SMA: smooth muscle actin
GPC3: glypican 3; HSP70-Heat shock protein 70
Hep Par 1: Hepatocyte paraffin 1
pCEA: polyclonal carcinoembryonic antigen
AFP: alpha-fetoprotein
PAK5: p21-activated kinase
HMGB1: high mobility group 1
miRNA/miR: microRNA
VEGF: vascular endothelial growth factor
Hif: hypoxia inducible factor
EMT: epithelial-mesenchymal transition
MMP: matrix metalloproteinases
CDK: cyclin-dependent kinases
DFS: disease-free survival
mTOR: mammalian target of rapamycin
EGF: epithelial growth factor
MDR: multidrug resistance
CCA: cholangiocarcinoma
ICC: intrahepatic cholangiocarcinoma
ECC: extrahepatic cholangiocarcinoma
PSC: primary sclerosing cholangitis
FISH: fluorescent in situ hybridization
qRT-PCR: quantitative real time polymerase chain reaction
FGF: fibroblast growth factor
HGF: hepatocyte growth factor
MAPK: mitogen-activated protein kinase
IDH1: isocitrate dehydrogenase 1
AFP: Alpha-fetoprotein
AFPL3: Lens culinaris agglutinin reactive AFP
AFU: a-l-fucosidase
BBTDs: benign biliary tract diseases
CA 19-9: carbohydrate antigen 19-9
CEA: carcinoembryonic antigen
CgA: Chromogranin A
CLIP: Cancer of the liver Italian program
CTC: circulating tumoral cells
CYFRA 21-1: cytokeratin-19 fragments
DCP: Des-γ-carboxy prothrombin
EGFR: Epidermal growth factor receptor
ELF: Embryonic Liver Fodrin
GP73: Golgi protein-73
HBV: hepatitis b virus
SNPs: single nucleotide polymorphisms

## References

[1] World Health Organization (2012). GLOBOCAN. Estimated Cancer Incidence, Mortality and Prevalence Worldwide in 2012.

[2] Parkin DM, Bray F, Ferlay J, Pisani P (2005). Global cancer statistics, 2002. CA Cancer J Clin.

[3] Qi X, Zhao Y, Li H, Guo X, Han G Management of hepatocellular carcinoma: An overview of major findings from meta-analyses. Oncotarget.

[4] Chen XP, Qiu FZ, Wu ZD, Zhang ZW, Huang ZY, Chen YF (2006). Long-term outcome of resection of large hepatocellular carcinoma. Br J Surg.

[5] Ruan DY, Lin ZX, Wang TT, Zhao H, Wu DH, Chen J, Dong M, Lin Q, Wu XY, Li Y Nomogram for preoperative estimation of long-term survival of patients who underwent curative resection with hepatocellular carcinoma beyond barcelona clinic liver cancer stage A1. Oncotarget.

[6] Di Benedetto F, Tarantino G, Ercolani G, Baccarani U, Montalti R, De Ruvo N, Berretta M, Adani GL, Zanello M, Tavio M, Cautero N, Tirelli U, Pinna AD, Gerunda GE, Guaraldi G (2013). Multicenter italian experience in liver transplantation for hepatocellular carcinoma in HIV-infected patients. Oncologist.

[7] Wang P, Li H, Shi B, Que W, Wang C, Fan J, Peng Z, Zhong L Prognostic factors in patients with recurrent hepatocellular carcinoma treated with salvage liver transplantation: a singlecenter study. Oncotarget.

[8] Yao FY, Ferrell L, Bass NM, Watson JJ, Bacchetti P, Venook A, Ascher NL, Roberts JP (2001). Liver transplantation for hepatocellular carcinoma: expansion of the tumor size limits does not adversely impact survival. Hepatology.

[9] Di Benedetto F, Berretta M, De Ruvo N, Tarantino G, D'Amico G, Ballarin R, Iemmolo RM, Gerunda GE (2012). Is advanced hepatocellular carcinoma amenable of cure by liver transplantation with sorafenib as a neoadjuvant approach plus m-TOR inhibitors monotherapy?. J Surg Oncol.

[10] Berretta M, Di Benedetto F, L Dal Maso, Cacopardo B, Nasti G, Facchini G, Bearz A, Spina M, Garlassi E, De Re V, Fiorica F, Lleshi A, Tirelli U (2013). Sorafenib for the treatment of unresectable hepatocellular carcinoma in HIV-positive patients. Anticancer Drugs.

[11] Berretta M, Stanzione B, Di Francia R, Tirelli U (2015). The expression of PD-L1 APE1 and P53 in hepatocellular carcinoma and its relationship to clinical pathology. Eur Rev Med Pharmacol Sci.

[12] Song PP, Xia JF, Inagaki Y, Hasegawa K, Sakamoto Y, Kokudo N, Tang W (2016). Controversies regarding and perspectives on clinical utility of biomarkers in hepatocellular carcinoma. World J Gastroenterol.

[13] Stanta G (2011). Guidelines for Molecular Analysis in Archive Tissues.

[14] International Consensus Group (2009). for Hepatocellular Neoplasia. Pathologic diagnosis of early hepatocellular carcinoma: A Report of the International Consensus Group for Hepatocellular Neoplasia. Hepatology.

[15] Di Tommaso L, Franchi G, Park YN, Fiamengo B, Destro A, Morenghi E, Montorsi M, Torzilli G, Tommasini M, Terracciano L, Tornillo L, Vecchione R, Roncalli M (2007). Diagnostic value of HSP70, glypican 3 and glutamine synthetase in hepatocellular nodules in cirrhosis. Hepatology.

[16] Canzonieri V, Alessandrini L, Caggiari L, Perin T, Berretta M, Valli De Re, Berretta M, Cacopardo B (2015). Clinico-pathological and molecular findings in hepatocellular carcinoma. Hepatocellular carcinoma in the 3rd millennium.

[17] Canzonieri V, Alessandrini L, Caggiari L, Perin T, Berretta M, Cannizzaro R, De Re V (2015). Hepatocellular carcinoma: an overview of clinico-pathological and molecular perspectives. WCRJ.

[18] Fang ZP, Jiang BG, Gu XF, Zhao B, Ge RL, Zhang FB (2014). P21- activated kinase 5 plays essential roles in the proliferation and tumorigenicity of human hepatocellular carcinoma. Acta Pharmacol Sin.

[19] Xiao J, Ding Y, Huang J, Li Q, Liu Y, Ni W, Zhang Y, Zhu Y, Chen L, Chen B (2014). The association of HMGB1 gene with the prognosis of HCC. PLoS One.

[20] Murakami Y, Yasuda T, Saigo K, Urashima T, Toyoda H, Okanoue T, Shimotohno K (2006). Comprehensive analysis of microRNA expression patterns in hepatocellular carcinoma and non-tumorous tissues. Oncogene.

[21] Toffanin S, Hoshida Y, Lachenmayer A, Villanueva A, Cabellos L, Minguez B, Savic R, Ward SC, Thung S, Chiang DY, Alsinet C, Tovar V, Roayaie S, Schwartz M, Bruix J, Waxman S, Friedman SL, Golub T, Mazzaferro V, Llovet JM MicroRNA-based classification of hepatocellular carcinoma and oncogenic role of miR-517a. Gastroenterology.

[22] Tanigawa N, Lu C, Mitsui T, Miura S (1997). Quantitation of sinusoidlike vessels in hepatocellular carcinoma: its clinical and prognostic significance. Hepatology.

[23] Nanashima A, Nakayama T, Sumida Y, Abo T, Takeshita H, Shibata K, Hidaka S, Sawai T, Yasutake T, Nagayasu T (2008). Relationship between microvessel count and post-hepatectomy survival in patients with hepatocellular carcinoma. World J Gastroenterol.

[24] Li X, Jiang J, Zhao X, Zhao Y, Cao Q, Zhao Q, Han H, Wang J, Yu Z, Peng B, Ying W, Qian X In-depth analysis of secretome and N-glycosecretome of human hepatocellular carcinoma metastatic cell lines shed light on metastasis correlated proteins. Oncotarget.

[25] Yang XR, Xu Y, Yu B, Zhou J, Qiu SJ, Shi GM, Zhang BH, Wu WZ, Shi YH, Wu B, Yang GH, Ji Y, Fan J (2010). High expression levels of putative hepatic stem/progenitor cell biomarkers related to tumour angiogenesis and poor prognosis of hepatocellular carcinoma. Gut.

[26] Yang P, Yuan W, He J, Wang J, Yu L, Jin X, Hu Y, Liao M, Chen Z, Zhang Y (2009). Overexpression of EphA2, MMP-9, and MVD-CD34 in hepatocellular carcinoma: Implications for tumor progression and prognosis. Hepatol Res.

[27] Rimassa L, Abbadessa G, Personeni N, Porta C, Borbath I, Daniele B, Salvagni S, Van Laethem JL, Van Vlierberghe H, Trojan J, De Toni EN, Weiss A, Miles S, Gasbarrini A, Lencioni M, Lamar ME, Wang Y, Shuster D, Schwartz BE, Santoro A Tumor and circulating biomarkers in patients with second-line hepatocellular carcinoma from the randomized phase II study with tivantinib. Oncotarget.

[28] Schoenleber SJ, Kurtz DM, Talwalkar JA, Roberts LR, Gores GJ Prognostic role of vascular endothelial growth factor in hepatocellular carcinoma: systematic review and meta-analysis. Br J Cancer.

[29] Xie H, Song J, Liu K, Ji H, Shen H, Hu S, Yang G, Du Y, Zou X, Jin H, Yan L, Liu J, Fan D (2008). The expression of hypoxia inducible factor-1alpha in hepatitis B virus-related hepatocellular carcinoma: correlation with patients’ prognosis and hepatitis B virus X protein. Dig Dis Sci.

[30] Bangoura G, Liu ZS, Qian Q, Jiang CQ, Yang GF, Jing S (2007). Prognostic significance of HIF-2alpha/EPAS1 expression in hepatocellular carcinoma. World J Gastroenterol.

[31] Zhang L, Huang G, Li X, Zhang Y, Jiang Y, Shen J, Liu J, Wang Q, Zhu J, Feng X, Dong J, Qian C (2013). Hypoxia induces epithelial-mesenchymal transition via activation of SNAI1 by hypoxia-inducible factor -1α in hepatocellular carcinoma. BMC Cancer.

[32] Yang MH, Chen CL, Chau GY, Chiou SH, Su CW, Chou TY, Peng WL, Wu JC (2009). Comprehensive analysis of the independent effect of twist and snail in promoting metastasis of hepatocellular carcinoma. Hepatology.

[33] Zhang CH, Xu GL, Jia WD, Li JS, Ma JL, Ren WH, Ge YS, Yu JH, Liu WB, Wang W (2012). Activation of STAT3 signal pathway correlates with twist and E-cadherin expression in hepatocellular carcinoma and their clinical significance. J Surg Res.

[34] Endo K, Ueda T, Ueyama J, Ohta T, Terada T (2000). Immunoreactive Ecadherin, alpha-catenin, beta-catenin, and gamma-catenin proteins in hepatocellular carcinoma: relationships with tumor grade, clinicopathologic parameters, and patients’ survival. Hum Pathol.

[35] Iso Y, Sawada T, Okada T, Kubota K (2005). Loss of E-cadherin mRNA and gain of osteopontin mRNA are useful markers for detecting early recurrence of HCV-related hepatocellular carcinoma. J Surg Oncol.

[36] Zhai B, Yan HX, Liu SQ, Chen L, Wu MC, Wang HY (2008). Reduced expression of E-cadherin/catenin complex in hepatocellular carcinomas. World J Gastroenterol.

[37] Na DC, Lee JE, Yoo JE, Oh BK, Choi GH, Park YN (2011). Invasion and EMT-associated genes are up-regulated in B viral hepatocellular carcinoma with high expression of CD133-human and cell culture study. Exp Mol Pathol.

[38] Cho SB, Lee KH, Lee JH, Park SY, Lee WS, Park CH, Kim HS, Choi SK, Rew JS (2008). Expression of E- and Ncadherin and clinicopathology in hepatocellular carcinoma. Pathol Int.

[39] Nart D, Yaman B, Yilmaz F, Zeytunlu M, Karasu Z, Kiliç M (2010). Expression of matrix metalloproteinase-9 in predicting prognosis of hepatocellular carcinoma after liver transplantation. Liver Transpl.

[40] Xu Y, Chen Q, Li W, Su X, Chen T, Liu Y, Zhao Y, Yu C (2010). Overexpression of transcriptional coactivator AIB1 promotes hepatocellular carcinoma progression by enhancing cell proliferation and invasiveness. Oncogene.

[41] Liu WB, Xu GL, Jia WD, Li JS, Ma JL, Chen K, Wang ZH, Ge YS, Ren WH, Yu JH, Wang W, Wang XJ (2011). Prognostic significance and mechanisms of patterned matrix vasculogenic mimicry in hepatocellular carcinoma. Med Oncol.

[42] Ip YC, Cheung ST, Fan ST (2007). Atypical localization of membrane type 1-matrix metalloproteinase in the nucleus is associated with aggressive features of hepatocellular carcinoma. Mol Carcinog.

[43] Yamamoto H, Itoh F, Adachi Y, Fukushima H, Itoh H, Sasaki S, Hinoda Y, Imai K (1999). Messenger RNA expression of matrix metalloproteinases and tissue inhibitors of metalloproteinases in human hepatocellular carcinoma. Jpn J Clin Oncol.

[44] Gorrin Rivas MJ, Arii S, Furutani M, Harada T, Mizumoto M, Nishiyama H, Fujita J, Imamura M (1998). Expression of human macrophage metalloelastase gene in hepatocellular carcinoma: correlation with angiostatin generation and its clinical significance. Hepatology.

[45] Matsuda Y, Ichida T, Genda T, Yamagiwa S, Aoyagi Y, Asakura H Loss of p16 contributes to p27 sequestration by cyclin D(1)-cyclin-dependent kinase 4 complexes and poor prognosis in hepatocellular carcinoma. Clin Cancer Res.

[46] Pinato DJ, Pirisi M, Maslen L, Sharma R Tissue biomarkers of prognostic significance in hepatocellular carcinoma. Adv Anat Pathol.

[47] Tannapfel A, Grund D, Katalinic A, Uhlmann D, Köckerling F, Haugwitz U, Wasner M, Hauss J, Engeland K, Wittekind C (2000). Decreased expression of p27 protein is associated with advanced tumor stage in hepatocellular carcinoma. Int J Cancer.

[48] Ito Y, Takeda T, Sakon M, Tsujimoto M, Monden M, Matsuura N (2001). Expression of p57/Kip2 protein in hepatocellular carcinoma. Oncology.

[49] Nakai S, Masaki T, Shiratori Y, Ohgi T, Morishita A, Kurokohchi K, Watanabe S, Kuriyama S (2002). Expression of p57(KIP2) in hepatocellular carcinoma: relationship between tumor differentiation and patient survival. Int J Oncol.

[50] Nan KJ, Guo H, Ruan ZP, Jing Z, Liu SX (2005). Expression of p57(kip2) and its relationship with clinicopathology, PCNA and p53 in primary hepatocellular carcinoma. World J Gastroenterol.

[51] Teramoto T, Satonaka K, Kitazawa S, Fujimori T, Hayashi K, Maeda S (1994). p53 gene abnormalities are closely related to hepatoviral infections and occur at a late stage of hepatocarcinogenesis. Cancer Res.

[52] Lee TK, Man K, Poon RT, Lo CM, Ng IO, Fan ST (2004). Disruption of p53-p21/ WAF1 cell cycle pathway contributes to progression and worse clinical outcome of hepatocellular carcinoma. Oncol Rep.

[53] Hu TH, Wang CC, Huang CC, Chen CL, Hung CH, Chen CH, Wang JH, Lu SN, Lee CM, Changchien CS, Tai MH (2007). Down-regulation of tumor suppressor gene PTEN, overexpression of p53, plus high proliferating cell nuclear antigen index predict poor patient outcome of hepatocellular carcinoma after resection. Oncol Rep.

[54] Yuan RH, Jeng YM, Hu RH, Lai PL, Lee PH, Cheng CC, Hsu HC (2011). Role of p53 and betacatenin mutations in conjunction with CK19 expression on early tumor recurrence and prognosis of hepatocellular carcinoma. J Gastrointest Surg.

[55] Honda K, Sbisa E, Tullo A, Papeo PA, Saccone C, Poole S, Pignatelli M, Mitry RR, Ding S, Isla A, Davies A, Habib NA (1998). p53 mutation is a poor prognostic indicator for survival in patients with hepatocellular carcinoma undergoing surgical tumour ablation. Br J Cancer.

[56] Park NH, Chung YH, Youn KH, Song BC, Yang SH, Kim JA, Lee HC, Yu E, Lee YS, Lee SG, Kim KW, Suh DJ (2001). Close correlation of p53 mutation to microvascular invasion in hepatocellular carcinoma. J Clin Gastroenterol.

[57] Sugo H, Takamori S, Kojima K, Beppu T, Futagawa S (1999). The significance of p53 mutations as an indicator of the biological behavior of recurrent hepatocellular carcinomas. Surg Today.

[58] Guo C, Liu QG, Zhang L, Song Tx, Yang X (2009). Expression and clinical significance of p53, JunB and KAI1/CD82 in human hepatocellular carcinoma. Hepatobiliary Pancreat Dis Int.

[59] Villanueva A, Chiang DY, Newell P, Peix J, Thung S, Alsinet C, Tovar V, Roayaie S, Minguez B, Sole M, Battiston C, Van Laarhoven S, Fiel MI, Di Feo A, Hoshida Y, Yea S, Toffanin S, Ramos A, Martignetti JA, Mazzaferro V, Bruix J, Waxman S, Schwartz M, Meyerson M, Friedman SL, Llovet JM (2008). Pivotal role of mTOR signaling in hepatocellular carcinoma. Gastroenterology.

[60] Zhu AX, Abrams TA, Miksad R, Blaszkowsky LS, Meyerhardt JA, Zheng H, Muzikansky A, Clark JW, Kwak EL, Schrag D, Jors KR, Fuchs CS, Iafrate AJ, Borger DR, Ryan DP (2011). Phase 1/2 study of everolimus in advanced hepatocellular carcinoma. Cancer.

[61] Ito Y, Takeda T, Sakon M, Tsujimoto M, Higashiyama S, Noda K, Miyoshi E, Monden M, Matsuura N (2001). Expression and clinical significance of erb-B receptor family in hepatocellular carcinoma. Br J Cancer.

[62] Zhao YN, Cao J, Wu FX, Ou C, Yuan WP, Mo QG, Wei W, Li Y, Su JJ, Liang AM (2004). Expression and significance of EGF mRNA and EGFR mRNA in hepatocellular carcinoma. Ai Zheng.

[63] Daveau M, Scotte M, A Fr François, Coulouarn C, Ros G, Tallet Y, Hiron M, Hellot MF, Salier JP (2003). Hepatocyte growth factor, transforming growth factor alpha, and their receptors as combined markers of prognosis in hepatocellular carcinoma. Mol Carcinog.

[64] Huang TJ, Huang BJ, Liang QW, Huang CW, Fang Y (2004). Dual fluorescence in situ hybridization in detection of HER-2 oncogene amplification in primary hepatocellular carcinoma. Hepatobiliary Pancreat Dis Int.

[65] Han Li-Li, Lv Yi, Guo Hui (2014). Zhi-Ping Ruan, Ke-Jun Nan. Implications of biomarkers in human hepatocellular carcinoma pathogenesis and therapy. World J Gastroenterol.

[66] Berretta S., Fisichella R., Spartà D., Lleshi A., Nasti G (2015). Primary liver cancer: clinical aspects, prognostic factors and predictive response to therapy. WCRJ.

[67] Abou-Alfa GK, Schwartz L, Ricci S, Amadori D, Santoro A, Figer A, De Greve J, Douillard JY, Lathia C, Schwartz B, Taylor I, Moscovici M, Saltz LB (2006). Phase II study of sorafenib in patients with advanced hepatocellular carcinoma. J Clin Oncol.

[68] Ozenne V, Paradis V, Pernot S, Castelnau C, Vullierme MP, Bouattour M, Valla D, Farges O, Degos F (2010). Tolerance and outcome of patients with unresectable hepatocellular carcinoma treated with sorafenib. Eur J Gastroenterol Hepatol.

[69] Chen D, Zhao P, Li SQ, Xiao WK, Yin XY, Peng BG, Liang LJ (2013). Prognostic impact of pERK in advanced hepatocellular carcinoma patients treated with sorafenib. Eur J Surg Oncol.

[70] Arao T, Ueshima K, Matsumoto K, Nagai T, Kimura H, Hagiwara S, Sakurai T, Haji S, Kanazawa A, Hidaka H, Iso Y, Kubota K, Shimada M, Utsunomiya T, Hirooka M, Hiasa Y, Toyoki Y, Hakamada K, Yasui K, Kumada T, Toyoda H, Sato S, Hisai H, Kuzuya T, Tsuchiya K, Izumi N, Arii S, Nishio K, Kudo M (2013). FGF3/ FGF4 amplification and multiple lung metastases in responders to sorafenib in hepatocellular carcinoma. Hepatology.

[71] Ahn SM, Jang SJ, Shim JH, Kim D, Hong SM, Sung CO, Baek D, Haq F, Ansari AA, Lee SY, Chun SM, Choi S, Choi HJ, Kim J, Kim S, Hwang S, Lee YJ, Lee JE, Jung WR, Jang HY, Yang E, Sung WK, Lee NP, Mao M, Lee C, Zucman-Rossi J, Yu E, Lee HC, Kong G (2014). Genomic portrait of resectable hepatocellular carcinomas: implications of RB1 and FGF19 aberrations for patient stratification. Hepatology.

[72] Wang K, Lim HY, Shi S, Lee J, Deng S, Xie T, Zhu Z, Wang Y, Pocalyko D, Yang WJ, Rejto PA, Mao M, Park CK, Xu J Genomic landscape of copy number aberrations enables the identification of oncogenic drivers in hepatocellular carcinoma. Hepatology.

[73] Chochi Y, Kawauchi S, Nakao M, Furuya T, Hashimoto K, Oga A, Oka M, Sasaki K (2009). A copy number gain of the 6p arm is linked with advanced hepatocellular carcinoma: an array-based comparative genomic hybridization study. J Pathol.

[74] Takeo S, Arai H, Kusano N, Harada T, Furuya T, Kawauchi S, Oga A, Hirano T, Yoshida T, Okita K, Sasaki K (2001). Examination of oncogene amplification by genomic DNA microarray in hepatocellular carcinomas: comparison with comparative genomic hybridization analysis. Cancer Genet Cytogenet.

[75] Nishida N, Fukuda Y, Komeda T, Kita R, Sando T, Furukawa M, Amenomori M, Shibagaki I, Nakao K, Ikenaga M (1994). Amplification and overexpression of the cyclin D1 gene in aggressive human hepatocellular carcinoma. Cancer Res.

[76] Giordano S, Columbano A (2013). MicroRNAs: New Tools for Diagnosis, Prognosis, and Therapy in Hepatocellular Carcinoma. Hepatology.

[77] Malaguarnera G, Giordano M, Paladina I, Berretta M, Cappellani A, Malaguarnera M (2010). Serum Markers of Hepatocellular Carcinoma. Dig Dis Sci.

[78] El-Serag HB, Marrero JA, Rudolph L, Reddy KR (2008). Diagnosis and treatment of hepatocellular carcinoma. Gastroenterology.

[79] Chang TS, Wu YC, Tung SY, Wei KL, Hsieh YY, Huang HC, Chen WM, Shen CH, Lu CH, Wu CS, Tsai YH, Huang YH (2015). Alpha- Fetoprotein Measurement Benefits Hepatocellular Carcinoma Surveillance in Patients with Cirrhosis. Am J Gastroenterol.

[80] Cho HJ, Seo YS, Lee KG, Hyun JJ, An H, Keum B, Kim JH, Yim J, Jeen YT, Lee HS, Chun HJ, Um SH, Kim CD, Ryu HS (2011). Serum aminotransferase levels instead of etiology affects the accuracy of transient elastography in chronic viral hepatitis patients. J Gastroenterol Hepatol.

[81] Chrysanthos NV, Papatheodoridis GV, Savvas S, Kafiri G, Petraki K, Manesis EK, Archimandritis AJ (2006). Aspartate aminotransferase to platelet ratio index for fibrosis evaluation in chronic viral hepatitis. Eur J Gastroenterol Hepatol.

[82] Degos F, Perez P, Roche B, Mahmoudi A, Asselineau J, Voitot H, Bedossa P (2010). Diagnostic accuracy of FibroScan and comparison to liver fibrosis biomarkers in chronic viral hepatitis: a multicenter prospective study (the FIBROSTIC study). J Hepatol.

[83] World Health Organization. Guidelines for the Prevention, Care and Treatment of Persons with Chronic Hepatitis B Infection. http://www.who.int/hiv/pub/hepatitis/hepatitis-b-guidelines/en/.

[84] Lee E, Edward S, Singal AG, Lavieri MS, Volk M (2013). Improving screening for hepatocellular carcinoma by incorporating data on levels of α-fetoprotein, over time. Clin Gastroenterol Hepatol.

[85] Singal AG, Conjeevaram HS, Volk ML, Fu S, Fontana RJ, Askari F, Su GL, Lok AS, Marrero JA (2012). Effectiveness of hepatocellular carcinoma surveillance in patients with cirrhosis. Cancer Epidemiol Biomarkers Prev.

[86] Sherman M (2010). Serological surveillance for hepatocellular carcinoma: time to quit. J Hepatol.

[87] Biondi A, Malaguarnera G, Vacante M, Berretta M, D'Agata V, Malaguarnera M, Basile F, Drago F, Bertino G (2012). Elevated serum levels of Chromogranin A in hepatocellular carcinoma. BMC Surgery.

[88] Mizejewski GJ (2002). Biological role of alpha-fetoprotein in cancer: prospects for anticancer therapy. Expert Rev Anticancer Ther.

[89] Singal A, Volk ML, Waljee A, Salgia R, Higgins P, Rogers MA, Marrero JA (2009). Meta-analysis: surveillance with ultrasound for early-stage hepatocellular carcinoma in patients with cirrhosis. Aliment Pharmacol Ther.

[90] Lok AS, Sterling RK, Everhart JE, Wright EC, Hoefs JC, Di Bisceglie AM, Morgan TR, Kim HY, Lee WM, Bonkovsky HL, Dienstag JL (2010). Des-gamma-carboxy prothrombin and alphafetoprotein as biomarkers for the early detection of hepatocellular carcinoma. Gastroenterology.

[91] Paul SB, Gulati MS, Sreenivas V, Madan K, Gupta AK, Mukhopadhyay S, Acharya SK (2007). Evaluating patients with cirrhosis for hepatocellular carcinoma: value of clinical symptomatology, imaging and alpha-fetoprotein. Oncology.

[92] Barletta E, Tinessa V, Daniele B (2005). Screening of hepatocellular carcinoma: role of the alpha-fetoprotein (AFP) and ultrasonography. Recenti Prog Med.

[93] Daniele B, Bencivenga A, Megna AS, Tinessa V (2004). Alpha-fetoprotein and ultrasonography screening for hepatocellular carcinoma. Gastroenterology.

[94] Marrero JA (2005). Screening tests for hepatocellular carcinoma. Clin Liver Dis.

[95] Di Bisceglie AM, Hoofnagle JH (1989). Elevations in serum alphafetoprotein levels in patients with chronic hepatitis. B. Cancer.

[96] Zhou XD, Tang ZY, Fan J, Zhou J, Wu ZQ, Qin LX, Ma ZC, Sun HC, Qiu SJ, Yu Y, Ren N, Ye QH, Wang L, Ye SL (2009). Intrahepatic cholangiocarcinoma: report of 272 patients compared with 5,829 patients with hepatocellular carcinoma. J Cancer Res Clin Oncol.

[97] Tateishi R, Yoshida H, Matsuyama Y, Mine N, Kondo Y, Omata M (2008). Diagnostic accuracy of tumor markers for hepatocellular carcinoma: a systematic review. Hepatol Int.

[98] Saffroy R, Pham P, Reffas M, Takka M, Lemoine A, Debuire B (2007). New perspectives and strategy research biomarkers for hepatocellular carcinoma. Clin Chem Lab Med.

[99] Bruix J, Sherman M, Llovet JM, Beaugrand M, Lencioni R, Burroughs AK, Christensen E, Pagliaro L, Colombo M, J; Rodés (2001). EASL Panel of Experts on HCC. Clinical management of hepatocellular carcinoma. Conclusions of the Barcelona-2000 EASL conference. European association for the study of the liver. J Hepatol.

[100] Han SJ, Yoo S, Choi SH, Hwang EH (1997). Actual half-life of alphafetoprotein as a prognostic tool in pediatric malignant tumors. Pediatr Surg Int.

[101] Oka H, Saito A, Ito K, Kumada T, Satomura S, Kasugai H, Osaki Y, Seki T, Kudo M, M; Tanaka (2001). Collaborative Hepato-Oncology Study Group of Japan. Multicenter prospective analysis of newly diagnosed hepatocellular carcinoma with respect to the percentage of Lens culinaris agglutinin-reactive alpha-fetoprotein. J Gastroenterol Hepatol.

[102] Sato Y, Nakata K, Kato Y, Shima M, Ishii N, Koji T, Taketa K, Endo Y, Nagataki S (1993). Early recognition of hepatocellular carcinoma based on altered profiles of alpha-fetoprotein. N Engl J Med.

[103] Aoyagi Y, Suzuki Y, Isemura M, Nomoto M, Sekine C, Igarashi K, Ichida F (1988). The fucosylation index of alpha-fetoprotein and its usefulness in the early diagnosis of hepatocellular carcinoma. Cancer.

[104] Taketa K (1990). Alpha-fetoprotein: reevaluation in hepatology. Hepatology.

[105] Kumada T, Toyoda H, Tada T, Kiriyama S, Tanikawa M, Hisanaga Y, Kanamori A, Tanaka J, Kagebayashi C, Satomura S (2014). High-sensitivity Lens culinaris agglutinin-reactive alpha-fetoprotein assay predicts early detection of hepatocellular carcinoma. J Gastroenterol.

[106] Miyaaki H, Nakashima O, Kurogi M, Eguchi K, Kojiro M (2007). Lens culinaris agglutinin-reactive alpha-fetoprotein and protein induced by vitamin K absence II are potential indicators of a poor prognosis: a histopathological study of surgically resected hepatocellular carcinoma. J Gastroenterol.

[107] Sassa T, Kumada T, Nakano S, Uematsu T (1999). Clinical utility of simultaneous measurement of serum high-sensitivity des-gammacarboxy prothrombin and Lens culinaris agglutinin A-reactive alpha-fetoprotein in patients with small hepatocellular carcinoma. Eur J Gastroenterol Hepatol.

[108] Hayashi K, Kumada T, Nakano S, Takeda I, Sugiyama K, Kiriyama S, Sone Y, Miyata A, Shimizu H, Satomura S (1999). Usefulness of measurement of Lens culinaris agglutinin-reactive fraction of alphafetoprotein as a marker of prognosis and recurrence of small hepatocellular carcinoma. Am J Gastroenterol.

[109] Yamashita F, Tanaka M, Satomura S, Tanikawa K (1995). Monitoring of lectin-reactive alpha-fetoproteins in patients with hepatocellular carcinoma treated using transcatheter arterial embolization. Eur J Gastroenterol Hepatol.

[110] Yamashiki N, Seki T, Wakabayashi M, Nakagawa T, Imamura M, Tamai T, Nishimura A, Inoue K, Okamura A, Arita S, Harada K (1999). Usefulness of Lens culinaris agglutinin A-reactive fraction of alpha-fetoprotein (AFP-L3) as a marker of distant metastasis from hepatocellular carcinoma. Oncol Rep.

[111] Ono M, Ohat H, Ohhira M, Sekiya C, Namiki M (1990). Measurement of immunoreactive prothrombin precursor and vitamin-K-dependent gammacarboxylation in human hepatocellular tissues: decreased carboxylation of prothrombin precursor as a cause of desgamma- carboxyprothrombin synthesis. Tumour Biol.

[112] Nakagawa T, Seki T, Shiro T, Wakabayashi M, Imamura M, Itoh T, Tamai T, Nishimura A, Yamashiki N, Matsuzaki K, Sakaida N, Inoue K, Okamura A (1999). Clinicopathologic significance of protein induced by vitamin k absence or antagonistic II and alpha-fetoprotein in hepatocellular carcinoma. Int J Oncol.

[113] Fujiyama S, Tanaka M, Maeda S, Ashihara H, Hirata R, Tomita K (2002). Tumor markers in early diagnosis, follow-up and management of patients with hepatocellular carcinoma. Oncology.

[114] Fujikawa T, Shiraha H, Ueda N, Takaoka N, Nakanishi Y, Matsuo N, Tanaka S, Nishina S, Suzuki M, Takaki A, Sakaguchi K, Shiratori Y (2007). Des-gamma-carboxyl prothrombin-promoted vascular endothelial cell proliferation and migration. J Biol Chem.

[115] Suehiro T, Sugimachi K, Matsumata T, Itasaka H, Taketomi A, Maeda T (1994). Protein induced by vitamin K absence or antagonist II (PIVKA-II) as a prognostic marker in hepatocellular carcinoma: comparison with a-fetoprotein. Cancer.

[116] Toyosaka A, Okamoto E, Mitsunobu M, Oriyama T, Nakao N, Miura K (1996). Intrahepatic metastases in hepatocellular carcinoma: evidence for spread via the portal vein as an efferent vessel. Am J Gastroenterol.

[117] Hakado S, Sakisaka S, Chayama K, Okanoue T, Toyoda J, Izumi N, Matsumoto A, Takehara T, Ido A, Hiasa Y, Yoshioka K, Nomura H, Ueno Y, Seike M, Kumada H (2015). Alpha-fetoprotein and des-gamma-carboxy-prothrombin at twenty-four weeks after interferon-based therapy predict hepatocellular carcinoma development. World J Hepatol.

[118] Zhu R, Yang J, Xu L, Dai W, Wang F, Shen M, Zhang Y, Zhang H, Chen K, Cheng P, Wang C, Zheng Y, Li J, Lu J, Zhou Y, Wu D, Guo C (2014). Diagnostic Performance of Des-γ-carboxy Prothrombin for Hepatocellular Carcinoma: A Meta-Analysis. Gastroenterol Res Pract.

[119] Bernfield M, Go¨tte M, Park PW, Reizes O, Fitzgerald ML, Lincecum J, Zako M (1999). Functions of cell surface heparan sulfate proteoglycans. Annu Rev Biochem.

[120] Song HH, Shi W, Filmus J (1997). OCI-5/rat glypican-3 binds to fibroblast growth factor-2 but not to insulin-like growth factor-2. J Biol Chem.

[121] Hsue HC, Cheng W, Lai Pl (1997). Cloning and expression of a developmentally regulated transcripts MXR7 in hepatocellular carcinoma: biological significance and temporospatial distribution. Cancer Res.

[122] Zhu ZW, Friess H, Wang L, Abou-Shady M, Zimmermann A, Lander AD, Korc M, Kleeff J, Büchler MW (2001). Enhanced glypican-3 expression differentiates the majority of hepatocellular carcinomas from benign hepatic disorders. Gut.

[123] Yukihiro H, Hiroaki K (2016). Glypican-3 is a prognostic factor and an immunotherapeutic target in hepatocellular carcinoma. World J Gastroenterol.

[124] Knapp LT, Klann E (2000). Superoxide- induced stimulation of protein kinase C via thiol modification and modulation of zinc content. J Biol Chem.

[125] Abou-Alfa GK, Puig O, Daniele B, Kudo M, Merle P, Park JW, Ross P, Peron JM, Ebert O, Chan S, Poon TP, Colombo M, Okusaka T, Ryoo BY, Minguez B, Tanaka T, Ohtomo T, Ukrainskyj S, Boisserie F, Rutman O, Chen YC, Xu C, Shochat E, Jukofsky L, Reis B, Chen G, Di Laurenzio L, Lee R, Yen CJ (2016). Randomized phase II placebo controlled study of codrituzumab in previously treated patients with advanced hepatocellular carcinoma. J Hepatol.

[126] Suzuki A, Hirata M, Kamimura K, Maniwa R, Yamanaka T, Mizuno K, Kishikawa M, Hirose H, Amano Y, Izumi N, Miwa Y, Ohno S (2004). aPKC acts upstream of PAR-1b in both the establishment and maintenance of mammalian epithelial polarity. Curr Biol.

[127] Regala RP, Weems C, Jamieson L, Copland JA, Thompson EA, Fields AP (2005). Atypical protein kinase C iota plays a critical role in human lung cancer cell growth and tumorigenicity. J Biol Chem.

[128] Wijnhoven BP, Dinjens WN, Pignatelli M (2000). E-cadherin-catenin cell-cell adhesion complex and human cancer. Br J Surg.

[129] Shiozaki H, Oka H, Inoue M, Tamura S, Monden M (1996). E-cadherin mediated adhesion system in cancer cells. Cancer.

[130] Hsu IC, Metcalf RA, Sun T, Welsh JA, Wang NJ, Harris CC (1991). Mutational hotspots in the p53 gene in human hepatocellular carcinomas. Nature.

[131] Liu S, Ma L, Huang W, Shai Y, Ji X, Ding L, Liu Y, Yu L, Zhao S (2006). Decreased expression of the human carbonyl reductase 2 Gene HCR2 in hepatocellular carcinoma. Cell Mol Biol Lett.

[132] Mise M, Arii S, Higashituji H, Furutani M, Furutani M, Niwano M, Harada T, Ishigami S, Toda Y, Nakayama H, Fukumoto M, Fujita J, Imamura M (1996). Clinical significance of vascular endothelial growth factor and basic fibroblast growth factor gene expression in liver tumor. Hepatology.

[133] Suzuki K, Hayashi M, Miyamaoto Y, Furutani M, Niwano M, Harada T, Ishigami S, Toda Y, Nakayama H, Fukumoto M, Fujita J, Imamura M (1996). Expression of vascular permeability factor/vascular endothelial growth factor in human hepatocellular carcinoma. Cancer Res.

[134] Mohle R, Green D, Moore MAS, Nachman RL, Rafii S (1997). Constitutive production and thrombin-induced release of vascular endothelial growth factor by human megakaryocytes and platelets. Proc Natl Acad Sci USA.

[135] Li XM, Tang ZY, Qin LX, Zhou J, Sun HC (1999). Serum vascular endothelial growth factor is a predictor of invasion and metastasis in hepatocellular carcinoma. J Exp Clin Cancer Res.

[136] Kong SY, Park JW, Lee JA, Park JE, Park KW, Hong EK, Kim CM (2007). Association between vascular endothelial growth factor gene polymorphisms and survival in hepatocellular carcinoma patients. Hepatology.

[137] Yvamoto EY, Ferreira RF, Nogueira V, Pinhe MA, Tenani GD, Andrade JG, Baitello ME, Gregório ML, Fucuta PS, Silva RF, Souza DR, Silva RC (2015). Influence of vascular endothelial growth factor and alpha-fetoprotein on hepatocellular carcinoma. Genet Mol Res.

[138] Zhan P, Qian Q, Yu LK (2013). Serum VEGF level is associated with the outcome of patients with hepatocellular carcinoma: a meta-analysis. Hepatobiliary Surg Nutr.

[139] Cao G, Li X, Qin C, Li J (2015). Prognostic Value of VEGF in Hepatocellular Carcinoma Patients Treated with Sorafenib: A Meta-Analysis. Med Sci Monit.

[140] Chinh Chung D, Thanh Long L, Nghia Son H, Tri Bao L, Minh Si D, Dong le V (2015). Downregulation of Vascular Endothelial Growth Factor Enhances Chemosensitivity by Induction of Apoptosis in Hepatocellular Carcinoma Cells. Cell J.

[141] Guan Q, Gu J, Zhang H, Ren W, Ji W, Fan Y (2015). Correlation between vascular endothelial growth factor levels and prognosis of hepatocellular carcinoma patients receiving radiofrequency ablation. Biotechnol Biotechnol Equip.

[142] Suminami Y, Kishi F, Sekiguchi K, Kato H (1991). Squamous cell carcinoma antigen is a new member of the serine protease inhibitors. Biochem Biophys Res Commun.

[143] Giannelli G, Marinosci F, Sgarra C, Lupo L, Dentico P, Antonaci S (2005). Clinical role of tissue and serum levels of SCCA antigen in hepatocellular carcinoma. Int J Cancer.

[144] Zhang J, Shao C, Zhou Q, Zhu Y, Zhu J, Tu C (2015). Diagnostic accuracy of serum squamous cell carcinoma antigen and squamous cell carcinoma antigen-immunoglobulin M for hepatocellular carcinoma: A meta-analysis. Mol Clin Oncol.

[145] Haidon GH, Hayes PC (1996). Screening for hepatocellular carcinoma. Eur J Gastroenterol Hepatol.

[146] Deugnier Y, David V, Bressot P, Mabo P, Delamaire D, Messner M, Bourel M, Legall JY (1984). Serum a-L-fucosidase: a new marker for the diagnosis of primary hepatic carcinoma?. Hepatology.

[147] Leray G, Deugnier Y, Jouanolle AM, Lehry D, Bretagne JF, Campion JP, Brissot P, Le Treut A (1989). Biochemical aspects of a-L-fucosidase in hepatocellular carcinoma. Hepatology.

[148] Giardina MG, Matarazzo M, Varriale A, Morante R, Napoli A, Martino R (1992). Serum alpha-L-fucosidase. A useful marker in the diagnosis of hepatocellular carcinoma. Cancer.

[149] Ishizuka H, Nakayama T, Matsuoka S, Gotoh I, Ogawa M, Suzuki K, Tanaka N, Tsubaki K, Ohkubo H, Arakawa Y, Okano T (1999). Prediction of the development of hepato-cellular-carcinoma in patients with liver cirrhosis by the serial determinations of serum alpha-L-fucosidase activity. Intern Med.

[150] Wang K, Guo W, Li N, Shi J, Zhang C, Lau WY, Wu M, Cheng S (2014). Alpha-1-fucosidase as a prognostic indicator for hepatocellular carcinoma following hepatectomy: a large-scale, long-term study. Br J Cancer.

[151] Bedossa P, Peltier E, Terries B, Franco D, Poynard T (1995). Transforming growth factor -b1 (TGF-b1) and TGF-b1 receptors in normal, cirrhotic and neoplastic human livers. Hepatology.

[152] Ito N, Kawata S, Tamura S, Takaishi K, Yabuuchi I, Matsuda Y, Nishioka M, Tarui S (1990). Expression of transforming growth factor b1 mRNA in human hepatocellular carcinoma. Jpn J Cancer Res.

[153] Mann CD, Neal CP, Garcea G, Manson MM, Dennison AR, Berry DP (2007). Prognostic molecular markers in hepatocellular carcinoma: a systematic review. Eur J Cancer.

[154] Ko TC, Tu W, Sakai T, Sheng H, Shao J, Beauchamp RD, Thompson EA (1998). TGF-b1 effects on proliferation of rat intestinal epithelial cells are due to inhibition of cyclin D1 expression. Oncogene.

[155] Izzo JG, Papadimitrakopoulou VA, Li XQ, Ibarguen H, Lee JS, Ro JY, El-Naggar A, Hong WK, Hittelman WN (1998). Dysregulated cyclin D1 expression early in head and neck tumorigenesis: in vivo evidence for an association with subsequent gene amplification. Oncogene.

[156] Seewaldt VL, Kim JH, Parker MB, Dietze EC, Vasan KV, Caldwell LE (1999). Dysregulated expression of cyclin D1 in normal human mammary epithelial cells inhibits all-trans-retinoic acidmediated G0/G1-phase arrest and differentiation in vitro. Exp Cell Res.

[157] Ji F, Fu SJ, Shen SL, Zhang LJ, Cao QH, Li SQ, Peng BG, Liang LJ, Hua YP (2015). The prognostic value of combined TGF-β1 and ELF in hepatocellular carcinoma. BMC Cancer.

[158] Kladney RD, Bulla GA, Guo L, Mason AL, Tollefson AE, Simon DJ, Koutoubi Z, Fimmel CJ (2000). GP73, a novel Golgi localized protein upregulated by viral infection. Gene.

[159] Kladney RD, Cui X, Bulla GA, Brunt EM, Fimmel CJ (2002). Expression of GP73, a resident membrane protein, in viral and non-viral liver disease. Hepatology.

[160] Block TM, Comunale MA, Lowman M, Steel LF, Romano PR, Fimmel C, Tennant BC, London WT, Evans AA, Blumberg BS, Dwek RA, Mattu TS, Mehta AS (2005). Use of targeted glycoproteins that correlated with liver cancer in woodchucks and humans. Proc Natl Acad Sci USA.

[161] Dai M, Chen X, Liu X, Peng Z, Meng J, Dai S (2015). Diagnostic Value of the Combination of Golgi Protein 73 and Alpha-Fetoprotein in Hepatocellular Carcinoma: A Meta-Analysis. PLoS One.

[162] Bröker ME, Ljzermans JN, Witjes CD, van Vuuren HJ, de Man RA (2014). The predictive value of Golgi protein 73 in differentiating benign from malignant liver tumors. PLoS One.

[163] Vogelstein B, Kinzler KW (1992). p53 function and dysfunction. Cell.

[164] Gannon JV, Greaves R, Iggo R, Lane DP (1990). Activating mutations in p53 produce a common conformational effect-a monoclonal antibody specific for the mutant form. EMBO J.

[165] Hsu H-C, Tseng H-J, Lai P-L, Lee P-H, Peng S-Y (1993). Expression of p53 gene in 184 unifocal hepatocellular carcinoma: association with tumor growth and invasiveness. Cancer Res.

[166] Hayashi H, Sugio K, Matsumata T, Adachi E, Takenaka K, Sugimachi K (1995). The clinical significance of p53 gene mutation in hepatocellular carcinomas from Japan. Hepatology.

[167] El Azm AR, Yousef M, Salah R, Mayah W, Tawfeek S, Ghorabah H, Mansour N (2013). Serum anti-P53 antibodies and alpha-fetoprotein in patients with non-B non-C hepatocellular carcinoma. Springerplus.

[168] Deftos LJ Chromogranin A: its role in endocrine function and as an endocrine and neuroendocrine tumor marker. Endocr Rev.

[169] Leone N, Pellicano R, Brunello F, Rizzetto M, Ponzetto A (2002). Elevated serum chromogranin A in patients with hepatocellular carcinoma. Clin Exp Med.

[170] Spadaro A, Ajello A, Morace C, Zirilli A, D'arrigo G, Luigiano C, Martino F, Bene A, Migliorato D, Turiano S, Ferraù O, Freni MA (2005). Serum chromogranin-A in hepatocellular carcinoma: diagnostic utility and limits.World. J Gastroenterol.

[171] Malaguarnera M, Vacante M, Fichera R, Cappellani A, Cristaldi E, Motta M (2010). Chromogranin A (CgA) serum level as a marker of progression in hepatocellular carcinoma (HCC) of elderly patients. Arch Gerontol Geriatr.

[172] Nakamura T (1994). Hepatocyte growth factor as mitogen, mitogen and morphogen and its roles in organ regeneration. Princess Takamatsu Symp.

[173] Birchmeier C, Gherardi E (1998). Development roles of HGF/SF and its receptor c-Met tyrosine kinase. Trends Cell Biol.

[174] Breuhan K, Longerich T, Schirmacher P (2006). Dysregulation of growth factor signalling in human hepatocellular carcinoma. Oncogene.

[175] Firtina Karagonlar Z, Koc D, Iscan E, Erdal E, Atabey N, Elevated HGF (2016). Expression as an Autocrine c-Met Activation Mechanism in Acquired Resistance to Sorafenib in HCC Cells. Cancer Sci.

[176] Mizuguchi T, Katsuramachi T, Nobuoka T, Kawamoto M, Oshima H, Kawasaki H, Kikuchi H, Shibata C, Hirata K (2004). Serum hyaluronate level for predicting subclinical liver dysfunction after hepatectomy. World J Surg.

[177] Wu FS, Zheng SS, Wu LJ, Ding W, Ma ZM, Wang ZM, Teng LS, Zhao WH (2006). Study on the prognostic value of hepatocyte growth factor and c-met for patients with hepatocellular carcinoma. Zhongua Wai Ke Za Zhi.

[178] Bothwell M (1995). Functional interactions of neurotrophins and neurotrophin receptors. Annu Rev Neurosci.

[179] Roux PP, Barker PA (2002). Neurotrophin signaling through the p75 neurotrophin receptor. Prog Neurobiol.

[180] Chapman BS (1995). A region of the 75-kDa neurotrophin receptor homologous to the death domains of TNFR-I and Fas. FEBS Lett.

[181] Tokusashi Y, Asai K, Tamakawa S, Yamamoto M, Yoshie M, Yaginuma Y, Miyokawa N, Aoki T, Kino S, Kasai S, Ogawa K (2005). Expression of NGF in hepatocellular carcinoma cells with its receptors in non-tumor cell components. Int J Cancer.

[182] Trim N, Morgan S, Evans M, Issa R, Fine D, Afford S, Wilkins B, Iredale J (2000). Hepatic stellate cells express the low affinity nerve growth factor receptor p75 and undergo apoptosis in response to nerve growth factor stimulation. Am J Pathol.

[183] Rasi G, Serafino A, Bellis L, Lonardo MT, Andreola F, Zonfrillo M, Vennarecci G, Pierimarchi P, P Sinibaldi Vallebona, Ettorre GM, Santoro E, Puoti C (2007). Nerve growth factor involvement in liver cirrhosis and hepatocellular carcinoma. World J Gastroenterol.

[184] Reddy SB, Patel T (2006). Current approaches to the diagnosis and treatment of cholangiocarcinoma. Curr Gastroenterol Rep.

[185] Blechacz B, Gores GJ (2008). Cholangiocarcinoma: advances in pathogenesis, diagnosis, and treatment. Hepatology.

[186] Clayton RA, Clarke DL, Currie EJ, Madhavan KK, Parks RW, Garden OJ (2003). Incidence of benign pathology in patients undergoing hepatic resection for suspected malignancy. Surgeon.

[187] Bangarulingam SY, Bjornsson E, Enders F, Barr Fritcher EG, Gores G, Halling KC, Lindor KD (2010). Long-term outcomes of positive fluorescence in situ hybridization tests in primary sclerosing cholangitis. Hepatology.

[188] Gonda TA, Glick MP, Sethi A, Poneros JM, Palmas W, Iqbal S, Gonzalez S, Nandula SV, Emond JC, Brown RS, Murty VV, Stevens PD (2012). Polysomy and p16 deletion by fluorescence in situ hybridization in the diagnosis of indeterminate biliary strictures. Gastrointest Endosc.

[189] Boldorini R, Paganotti A, Andorno S, Orlando S, Mercalli F, Orsello M, Ballarè M, Magnani C, Sartori M (2015). A multistep cytological approach for patients with jaundice and biliary strictures of indeterminate origin. J Clin Pathol.

[190] Eaton JE, Barr Fritcher EG, Gores GJ, Atkinson EJ, Tabibian JH, Topazian MD, Gossard AA, Halling KC, Kipp BR, Lazaridis KN (2015). Biliary multifocal chromosomal polysomy and cholangiocarcinoma in primary sclerosing cholangitis. Am J Gastroenterol.

[191] Ponsioen CY, Vrouenraets SM, van Milligen de Wit AW, Sturm P, Tascilar M, Offerhaus GJ, Prins M, Huibregtse K, Tytgat GN (1999). Value of brush cytology for dominant strictures in primary sclerosing cholangitis. Endoscopy.

[192] Andresen K, Boberg KM, Vedeld HM, Honne H, Jebsen P, Hektoen M, Wadsworth CA, Clausen OP, Lundin KE, Paulsen V, Foss A, Mathisen Ø, Aabakken L, Schrumpf E, Lothe RA, Lind GE (2015). Four DNA methylation biomarkers in biliary brush samples accurately identify the presence of cholangiocarcinoma. Hepatology.

[193] Kawahigashi Y, Mishima T, Mizuguchi Y, Arima Y, Yokomuro S, Kanda T, Ishibashi O, Yoshida H, Tajiri T, Takizawa T (2009). MicroRNA profiling of human intrahepatic cholangiocarcinoma cell lines reveals biliary epithelial cell-specific microRNAs. J Nippon Med Sch.

[194] Chen L, Yan HX, Yang W, Hu L, Yu LX, Liu Q, Li L, Huang DD, Ding J, Shen F, Zhou WP, Wu MC, Wang HY (2009). The role of microRNA expression pattern in human intrahepatic cholangiocarcinoma. J Hepatol.

[195] Collins AL, Wojcik S, Liu J, Frankel WL, Alder H, Yu L, Schmittgen TD, Croce CM, Bloomston M (2014). A differential microRNA profile distinguishes cholangiocarcinoma from pancreatic adenocarcinoma. Ann Surg Oncol.

[196] Silakit R, Loilome W, Yongvanit P, Chusorn P, Techasen A, Boonmars T, Khuntikeo N, Chamadol N, Pairojkul C, Namwat N (2014). Circulating miR-192 in liver fluke-associated cholangiocarcinoma patients: a prospective prognostic indicator. J Hepatobiliary Pancreat Sci.

[197] Zhang MY, Li SH, Huang GL, Lin GH, Shuang ZY, Lao XM, Xu L, Lin XJ, Wang HY, Li SP (2015). Identification of a novel microRNA signature associated with intrahepatic cholangiocarcinoma (ICC) patient prognosis. BMC Cancer.

[198] McNally ME, Collins A, Wojcik SE, Liu J, Henry JC, Jiang J, Schmittgen T, Bloomston M (2013). Concomitant dysregulation of microRNAs miR-151-3p and miR-126 correlates with improved survival in resected cholangiocarcinoma. HPB (Oxford).

[199] Chusorn P, Namwat N, Loilome W, Techasen A, Pairojkul C, Khuntikeo N, Dechakhamphu A, Talabnin C, Chan-On W, Ong CK, Teh BT, Yongvanit P (2013). Overexpression of microRNA-21 regulating PDCD4 during tumorigenesis of liver fluke-associated cholangiocarcinoma contributes to tumor growth and metastasis. Tumour Biol.

[200] Li B, Han Q, Zhu Y, Yu Y, Wang J, Jiang X (2012). Down-regulation of miR-214 contributes to intrahepatic cholangiocarcinoma metastasisby targeting Twist. FEBS J.

[201] Chang YT, Chang MC, Huang KW, Tung CC, Hsu C, Wong JM (2014). Clinicopathological and prognostic significances of EGFR, KRAS and BRAF mutations in biliary tract carcinomas in Taiwan. J Gastroenterol Hepatol.

[202] Yoshikawa D, Ojima H, Iwasaki M, Hiraoka N, Kosuge T, Kasai S, Hirohashi S, Shibata T (2008). Clinicopathological and prognostic significance of EGFR, VEGF, and HER2 expression in cholangiocarcinoma. Br J Cancer.

[203] Nitta T, Mitsuhashi T, Hatanaka Y, Miyamoto M, Oba K, Tsuchikawa T, Suzuki Y, Hatanaka KC, Hirano S, Matsuno Y (2014). Prognostic significance of epithelial-mesenchymal transition-related markers in extrahepatic cholangiocarcinoma: comprenhensive immunohistochemical study using a tissue microarray. Br J Cancer.

[204] Sirica AE (2008). Role of ErbB family receptor tyrosine kinases in intrahepatic cholangiocarcinoma. World J Gastroenterol.

[205] Graham RP, Barr Fritcher EG, Pestova E, Schulz J, Sitailo LA, Vasmatzis G, Murphy SJ, McWilliams RR, Hart SN, Halling KC, Roberts LR, Gores GJ, Couch FJ, Zhang L, Borad MJ, Kipp BR (2014). Fibroblast growth factor receptor 2 translocations in intrahepatic cholangiocarcinoma. Hum Pathol.

[206] Rizvi S, Borad MJ, Patel T, Gores GJ (2014). Cholangiocarcinoma: molecular pathways and therapeutic opportunities. Semin Liver Dis.

[207] Andersen JB, Spee B, Blechacz BR, Avital I, Komuta M, Barbour A, Conner EA, Gillen MC, Roskams T, Roberts LR, Factor VM, Thorgeirsson SS (2012). Genomic and genetic characterization of cholangiocarcinoma identifies therapeutic targets for tyrosine kinase inhibitors. Gastroenterology.

[208] Sia D, Hoshida Y, Villanueva A, Roayaie S, Ferrer J, Tabak B, Peix J, Sole M, Tovar V, Alsinet C, Cornella H, Klotzle B, Fan JB, Cotsoglou C, Thung SN, Fuster J, Waxman S, Garcia-Valdecasas JC, Bruix J, Schwartz ME, Beroukhim R, Mazzaferro V, Llovet JM (2013). Integrative molecular analysis of intrahepatic cholangiocarcinoma reveals 2 classes that have different outcomes. Gastroenterology.

[209] Arai Y, Totoki Y, Hosoda F, Shirota T, Hama N, Nakamura H, Ojima H, Furuta K, Shimada K, Okusaka T, Kosuge T, Shibata T (2014). Fibroblast growth factor receptor 2 tyrosine kinase fusions define a unique molecular subtype of cholangiocarcinoma. Hepatology.

[210] Wu YM, Su F, Kalyana-Sundaram S, Khazanov N, Ateeq B, Cao X, Lonigro RJ, Vats P, Wang R, Lin SF, Cheng AJ, Kunju LP, Siddiqui J, Tomlins SA, Wyngaard P, Sadis S, Roychowdhury S, Hussain MH, Feng FY, Zalupski MM, Talpaz M, Pienta KJ, Rhodes DR, Robinson DR, Chinnaiyan AM (2013). Identification of targetable FGFR gene fusions in diverse cancers. Cancer Discov.

[211] Voss JS, Holtegaard LM, Kerr SE, Fritcher EG, Roberts LR, Gores GJ, Zhang J, Highsmith WE, Halling KC, Kipp BR (2013). Molecular profiling of cholangiocarcinoma shows potential for targeted therapy treatment decisions. Hum Pathol.

[212] Jiao Y, Pawlik TM, Anders RA, Selaru FM, Streppel MM, Lucas DJ, Niknafs N, Guthrie VB, Maitra A, Argani P, Offerhaus GJ, Roa JC, Roberts LR, Gores GJ, Popescu I, Alexandrescu ST, Dima S, Fassan M, Simbolo M, Mafficini A, Capelli P, Lawlor RT, Ruzzenente A, Guglielmi A, Tortora G, de Braud F, Scarpa A, Jarnagin W, Klimstra D, Karchin R, Velculescu VE, Hruban RH, Vogelstein B, Kinzler KW, Papadopoulos N, Wood LD (2013). Exome sequencing identifies frequent inactivating mutations in BAP1, ARID1A and PBRM1 in intrahepatic cholangiocarcinomas. Nat Genet.

[213] Borger DR, Tanabe KK, Fan KC, Lopez HU, Fantin VR, Straley KS, Schenkein DP, Hezel AF, Ancukiewicz M, Liebman HM, Kwak EL, Clark JW, Ryan DP, Deshpande V, Dias-Santagata D, Ellisen LW, Zhu AX, Iafrate AJ (2012). Frequent mutation of isocitrate dehydrogenase (IDH)1 and IDH2 in cholangiocarcinoma identified through broad-based tumor genotyping. Oncologist.

[214] Kipp BR, Voss JS, Kerr SE, Barr Fritcher EG, Graham RP, Zhang L, Highsmith WE, Zhang J, Roberts LR, Gores GJ, Halling KC (2012). Isocitrate dehydrogenase 1 and 2 mutations in cholangiocarcinoma. Hum Pathol.

[215] Xu W, Yang H, Liu Y, Yang Y, Wang P, Kim SH, Ito S, Yang C, Wang P, Xiao MT, Liu LX, Jiang WQ, Liu J, Zhang JY, Wang B, Frye S, Zhang Y, Xu YH, Lei QY, Guan KL, Zhao SM, Xiong Y (2011). Oncometabolite 2-hydroxyglutarate is a competitive inhibitor of α-ketoglutarate-dependent dioxygenases. Cancer Cell.

[216] Wiggers JK, Ruys AT, Groot Koerkamp B, Beuers U, ten Kate FJ, van Gulik TM (2014). Differences in immunohistochemical biomarkers between intra- and extrahepatic cholangiocarcinoma: A systematic review and meta-analysis. J Gastroenterol Hepatol.

[217] Siegel R, Naishadham D, Jemal A (2012). Cancer statistics, 2012. CA Cancer J Clin.

[218] Hummel R, Hussey DJ, Haier J (2010). MicroRNAs: predictors and modifiers of chemo- and radiotherapy in different tumour types. Eur J Cancer (Oxford, England:1990).

[219] Meng F, Henson R, Lang M, Wehbe H, Maheshwari S, Mendell JT, Jiang J, Schmittgen TD, Patel T (2006). Involvement of human micro-RNA in growth and response to chemotherapy in human cholangiocarcinoma cell lines. Gastroenterology.

[220] Okamoto K, Miyoshi K, Murawaki Y (2013). miR-29b, miR-205 and miR-221 enhance chemosensitivity to gemcitabine in HuH28 human cholangiocarcinoma cells. PLOS ONE.

[221] Viterbo D, Gausman V, Gonda T (2016). Diagnostic and therapeutic biomarkers in pancreaticobiliary malignancy. World J Gastrointest Endosc.

[222] Uenishi T, Yamazaki O, Tanaka H, Takemura S, Yamamoto T, Tanaka S, Nishiguchi S, Kubo S (2008). Serum cytokeratin 19 fragment (CYFRA21-1) as a prognostic factor in intrahepatic cholangiocarcinoma. Ann Surg Oncol.

[223] Björnsson E, Kilander A, Olsson R (1999). CA 19-9 and CEA are unreliable markers for cholangiocarcinoma in patients with primary sclerosing cholangitis. Liver.

[224] Qin XL, Wang ZR, Shi JS, Lu M, Wang L, He QR (2004). Utility of serum CA19-9 in diagnosis of cholangiocarcinoma: in comparison with CEA. World J Gastroenterol.

[225] Charatcharoenwitthaya P, Enders FB, Halling KC, Lindor KD (2008). Utility of serum tumor markers, imaging, and biliary cytology for detecting cholangiocarcinoma in primary sclerosing cholangitis. Hepatology.

[226] Patel AH, Harnois DM, Klee GG, LaRusso NF, Gores GJ (2000). The utility of CA 19-9 in the diagnoses of cholangiocarcinoma in patients without primary sclerosing cholangitis. Am J Gastroenterol.

[227] Lumachi F, Lo Re G, Tozzoli R, D'Aurizio F, Facomer F, Chiara GB, Basso SM (2014). Measurement of serum carcinoembryonic antigen, carbohydrate antigen 19-9, cytokeratin-19 fragment and matrix metalloproteinase-7 for detecting cholangiocarcinoma: a preliminary case-control study. Anticancer Res.

[228] Chapman MH, Sandanayake NS, Andreola F, Dhar DK, Webster GJ, Dooley JS, Pereira SP (2011). Circulating CYFRA 21-1 is a Specific Diagnostic and Prognostic Biomarker in Biliary Tract Cancer. J Clin Exp Hepatol.

[229] Goydos JS, Brumfield AM, Frezza E, Booth A, Lotze MT, Carty E (1998). Marked elevation of serum interleukin-6 in patients with cholangiocarcinoma: validation of utility as a clinical marker. Ann Surg.

[230] Matsumoto K, Fujii H, Michalopoulos G, Fung JJ, Demetris AJ (1994). Human biliary epithelial cells secrete and respond to cytokines and hepatocyte growth factors in vitro: interleukin-6, hepatocyte growth factor and epidermal growth factor promote DNA synthesis in vitro. Hepatology.

[231] Bonney GK, Craven RA, Prasad R, Melcher AF, Selby PJ, Banks RE (2008). Circulating markers of biliary malignancy: opportunities in proteomics?. Lancet Oncol.

[232] Shen J, Wang W, Wu J, Feng B, Chen W, Wang M, Tang J, Wang F, Cheng F, Pu L, Tang Q, Wang X, Li X (2012). Comparative proteomic profiling of human bile reveals SSP411 as a novel biomarker of cholangiocarcinoma. PLoS One.

[233] Mitchell PS, Parkin RK, Kroh EM, Fritz BR, Wyman SK, Pogosova-Agadjanyan EL, Peterson A, Noteboom J, O'Briant KC, Allen A, Lin DW, Urban N, Drescher CW, Knudsen BS, Stirewalt DL, Gentleman R, Vessella RL, Nelson PS, Martin DB, Tewari M (2008). Circulating microRNAs as stable blood-based markers for cancer detection. Proc Natl Acad Sci USA.

[234] Meng F, Henson R, Lang M, Wehbe H, Maheshwari S, Mendell JT, Jiang J, Schmittgen TD, Patel T (2006). Involvement of human micro-RNA in growth and response to chemotherapy in human cholangiocarcinoma cell lines. Gastroenterology.

[235] Chen L, Yan HX, Yang W, Hu L, Yu LX, Liu Q, Li L, Huang DD, Ding J, Shen F, Zhou WP, Wu MC, Wang HY (2009). The role of microRNA expression pattern in human intrahepatic cholangiocarcinoma. J Hepatol.

[236] Karakatsanis A, Papaconstantinou I, Gazouli M, Lyberopoulou A, Polymeneas G, Voros D (2013). Expression of microRNAs, miR-21, miR-31, miR-122, miR-145, miR-146a, miR-200c, miR-221, miR-222, and miR-223 in patients with hepatocellular carcinoma or intrahepatic cholangiocarcinoma and its prognostic significance. Mol Carcinog.

[237] Liu CZ, Liu W, Zheng Y, Su JM, Li JJ, Yu L, He XD, Chen SS (2012). PTEN and PDCD4 are bona fide targets of microRNA-21 in human cholangiocarcinoma. Chin Med Sci J.

[238] Chusorn P, Namwat N, Loilome W, Techasen A, Pairojkul C, Khuntikeo N, Dechakhamphu A, Talabnin C, Chan-On W, Ong CK, Teh BT, Yongvanit P (2013). Overexpression of microRNA-21 regulating PDCD4 during tumorigenesis of liver fluke-associated cholangiocarcinoma contributes to tumor growth and metastasis. Tumour Biol.

[239] Yang JD, Campion MB, Liu MC, Chaiteerakij R, Giama NH, Ahmed Mohammed H, Zhang X, Hu C, Campion VL, Jen J, Venkatesh SK, Halling KC, Kipp BR, Roberts LR (2016). Circulating tumor cells are associated with poor overall survival in patients with cholangiocarcinoma. Hepatology.

[240] Al Ustwani O, Iancu D, Yacoub R, Iyer R (2012). Detection of circulating tumor cells in cancers of biliary origin. J Gastrointest Oncol.

[241] Janvilisri T, Leelawat K, Roytrakul S, Paemanee A, Tohtong R (2015). Novel Serum Biomarkers to Differentiate Cholangiocarcinoma from Benign Biliary Tract Diseases Using a Proteomic Approach. Dis Markers.

[242] Zhang MY, Li SH, Huang GL, Lin GH, Shuang ZY, Lao XM, Xu L, Lin XJ, Wang HY, Li SP (2015). Identification of a novel microRNA signature associated with intrahepatic cholangiocarcinoma (ICC) patient prognosis. BMC Cancer.

[243] Martinetti D, Costanzo R, Kadare S, Alimehmeti M, Colarossi C, Canzonieri V, Berretta M, Memeo L (2014). KRAS and BRAF mutational status in colon cancer from Albanian patients. Diagn Pathol.

[244] Chang YT, Chang MC, Huang KW, Tung CC, Hsu C, Wong JM (2014). Clinicopathological and prognostic significances of EGFR, KRAS and BRAF mutations in biliary tract carcinomas in Taiwan. J Gastroenterol Hepatol.

[245] Yoshikawa D, Ojima H, Iwasaki M, Hiraoka N, Kosuge T, Kasai S, Hirohashi S, Shibata T (2008). Clinicopathological and prognostic significance of EGFR, VEGF, and HER2 expression in cholangiocarcinoma. Br J Cancer.

[246] Leone F, Cavalloni G, Pignochino Y, Sarotto I, Ferraris R, Piacibello W, Venesio T, Capussotti L, Risio M, Aglietta M (2006). Somatic mutations of epidermal growth factor receptor in bile duct and gallbladder carcinoma. Clin Cancer Res.

[247] Gwak GY, Yoon JH, Shin CM, Ahn YJ, Chung JK, Kim YA, Kim TY, Lee HS (2005). Detection of response-predicting mutations in the kinase domain of the epidermal growth factor receptor gene in cholangiocarcinomas. J Cancer Res Clin Oncol.

[248] Philip PA, Mahoney MR, Allmer C, Thomas J, Pitot HC, Kim G, Donehower RC, Fitch T, Picus J, Erlichman C (2006). Phase II study of erlotinib in patients with advanced biliary cancer. J Clin Oncol.

[249] Viloria-Petit A, Crombet T, Jothy S, Hicklin D, Bohlen P, Schlaeppi JM, Rak J, Kerbel RS (2001). Acquired resistance to the antitumor effect of epidermal growth factor receptor-blocking antibodies in vivo: a role or altered tumor angiogenesis. Cancer Res.

[250] Yoshikawa D, Ojima H, Kokubu A, Ochiya T, Kasai S, Hirohashi S, Shibata T (2009). Vandetanib (ZD6474), an inhibitor of VEGFR and EGFR signalling, as a novel molecular-targeted therapy against cholangiocarcinoma. Br J Cancer.

[251] Voss JS, Holtegaard LM, Kerr SE, Fritcher EG, Roberts LR, Gores J, Zhang J, Highsmith WE, Halling KC, Kipp BR (2013). Molecular profiling of cholangiocarcinoma shows potential for targeted therapy treatment decisions. Hum Pathol.

[252] Tannapfel A, Sommerer F, Benicke M, Katalinic A, Uhlmann D, Witzigmann H, Hauss J, Wittekind C (2003). Mutations of the BRAF gene in cholangiocarcinoma but not in hepatocellular carcinoma. Gut.

[253] Robertson S, Hyder O, Dodson R, Nayar SK, Poling J, Beierl K, Eshleman JR, Lin MT, Pawlik TM, Anders RA (2013). The frequency of KRAS and BRAF mutations in intrahepatic cholangiocarcinomas and their correlation with clinical outcome. Hum Pathol.

[254] Altimari A, Fiorentino M, Gabusi E, Gruppioni E, Corti B, D'Errico A, Grigioni WF (2003). Investigation of ErbB1 and ErbB2 expression for therapeutic targeting in primary liver tumours. Dig Liver Dis.

[255] Law LY (2012). Dramatic response to trastuzumab and paclitaxel in a patient with human epidermal growth factor receptor 2-positive metastatic cholangiocarcinoma. J Clin Oncol.

[256] Okamoto K, Miyoshi K, Murawaki Y (2013). miR-29b, miR-205 and miR-221 enhance chemosensitivity to gemcitabine in HuH28 human cholangiocarcinoma cells. PLoS One.

[257] Olaru AV, Ghiaur G, Yamanaka S, Luvsanjav D, An F, Popescu I, Alexandrescu S, Allen S, Pawlik TM, Torbenson M, Georgiades C, Roberts LR, Gores GJ, Ferguson-Smith A, Almeida MI, Calin GA, Mezey E, Selaru FM (2011). MicroRNA down-regulated in human cholangiocarcinoma control cell cycle through multiple targets involved in the G1/S checkpoint. Hepatology.

[258] De Re V, Caggiari L, De Zorzi M, Repetto O, Zignego AL, Izzo F, Tornesello ML, Buonaguro FM, Mangia A, Sansonno D, Racanelli V, De Vita S, Pioltelli P, Vaccher E, Berretta M, Mazzaro C, Libra M, Gini A, Zucchetto A, Cannizzaro R, De Paoli P (2015). Genetic diversity of the KIR/HLA system and susceptibility to hepatitis C virus-related diseases. PLoS One.

[259] De Re V, Gragnani L, Fognani E, Piluso A, Izzo F, Mangia A, Crovatto M, Gava G, Casarin P, Sansonno D, Racanelli V, De Vita S, Pioltelli P, Caggiari L, De Zorzi M, Berretta M, Gini A, Zucchetto A, Buonaguro FM, De Paoli P, Zignego AL (2014). Impact of immunogenetic IL28B polymorphism on natural outcome of HCV infection. Biomed Res Int.

